# Engineering a robust *Is*PETase for energy-efficient PET depolymerization in natural seawater at ambient temperatures

**DOI:** 10.1007/s44307-026-00104-z

**Published:** 2026-04-15

**Authors:** Xin Huang, Qian Jia, Guang Li, Xiangpeng Yang, Shujing Xu, Jianzhong Liu, Wenjun Li, Yuhuan Liu, Wei Xie, Lichuang Cao

**Affiliations:** State Key Laboratory of Biocontrol, Innovation Center for Evolutionary Synthetic Biology, Guangzhou Innovation Center of Biotechnology and Biomanufacturing, School of Life Sciences, Sun Yat-Sen University, Guangzhou, 510275 China

**Keywords:** PET hydrolase, Bio-recycling, Enzymatic depolymerization, Enzyme engineering, Seawater bio-catalysis, Simultaneous enzymatic depolymerization and fermentation (SEDF)

## Abstract

Enzymatic depolymerization of polyethylene terephthalate (PET), the world’s most widely used polyester, in seawater at ambient temperature offers a promising and energy-efficient route for freshwater-free plastic recycling. While a number of PET hydrolases have been reported in recent years, their potential under saline conditions remains largely unexplored. Here, we screened eight enzymes in artificial seawater at 30 °C and engineered the most active one, *Is*PETase, using a semi-rational strategy focused on rigidifying flexible sites. The resulting variant M8 showed simultaneous enhancementsin thermostability (ΔT_m_ = + 27.3 °C), activity (1.14-fold increase) and soluble expression yield (14.3-fold increase). The overall depolymerization efficiency of M8 surpassed that of the thermostable benchmark enzymes DuraPETase and LCC-ICCG by 32.2- and 10.4-fold, respectively. Notably, M8 achieved continuous and efficient depolymerization of 15% (w/v) PET powder in natural seawater at 37 °C, yielding monomers at a rate of 15.4 mM/day, a concentration sufficient to support downstream bacterial assimilation. This work provides an efficient enzymatic platform and paves the way for fully integrated, seawater-based plastic bioconversion processes.

**Supplementary Information:**

The online version contains supplementary material available at 10.1007/s44307-026-00104-z.

## Introduction

Plastic pollution has become a severe challenge to the global environmental sustainability and public health (Cottom et al. 2024; Pottinger et al. 2024). Poly(ethylene terephthalate) (PET), a high molecular weight polymer composed of terephthalic acid (TPA) and ethylene glycol (EG), has been extensively used in packaging and textiles due to its excellent durability, light weight and plasticity (Taniguchi et al. 2019). Annual global production of PET exceeds 80 million metric tons (Mican et al. 2024), making it the most widely demanded polyester and a major contributor to the plastic pollution crisis (Cowger et al. 2024). Conventional mechanical and chemical recycling methods are often hampered by high energy costs, substantial freshwater consumption, and the downcycling of material quality (Ragaert et al. 2017). In contrast, enzymatic depolymerization offers a promising green alternative by enabling the selective and mild breakdown of PET into its constituent monomers (Tournier et al. 2023). These monomers can not only be repolymerized into virgin-quality plastics but also serve as building blocks for producing higher-value chemicals through synthetic biology approaches, significantly enhancing the economic potential, and enabling a closed-loop, sustainable system for plastic management (Liu et al. 2025a; Wang et al. 2025a; Zhou et al. 2025b).

Extensive research conducted over the past two decades has identified many PET-hydrolyzing enzymes and enhanced their performance through protein engineering (Liu et al. 2025b). Current mainstream enzymatic depolymerization processes predominantly depend on thermostable enzymes and operate near the glass transition temperature (T_g_) of PET (65–75 °C) in artificial buffer systems (Zhou et al. 2025a). The elevated temperature increases the flexibility of PET polymer chains and substrate accessibility, while also promoting enzymatic hydrolysis kinetics following the Arrhenius equation (Wei et al. 2022). However, this high-temperature strategy incurs from substantial energy consumption and freshwater reliance. A further downstream incompatibility arises since most monomer-assimilating microbes and synthetic biology chassis organisms grow optimally at 30–40 °C (Brandenberg et al. 2022; Narancic et al. 2021; Tiso et al. 2021; Werner et al. 2021), necessitating an energy-intensive cooling step before microbial conversion can begin. In contrast, performing enzymatic depolymerization directly in seawater at lower temperatures presents a promising alternative, as it would drastically reduce​ energy input, eliminate freshwater use, and enable seamless integration with downstream biological upcycling. This approach aligns perfectly with the goals of next-generation industrial biotechnology (NGIB) by providing a pathway to​ continuous, non-sterile, and sustainable bioprocessing (Chen and Jiang 2018; Tan et al. 2021; Ye et al. 2023). Consequently, developing efficient enzymes that exhibit high activity and stability under ambient-temperature and high-salinity conditions, coupled with high-yield soluble expression, has become a pivotal prerequisite. Despite the availability of many PET hydrolases, their potential under such physiologically relevant and industrially applicable environments remains severely underexplored.

In this study, we report a proof-of-concept for enzymatic PET depolymerization in seawater at ambient temperature using an engineered hydrolase. We first screened previously reported PET hydrolases in the artificial seawater to identify a promising candidate. This enzyme was subsequently engineered to simultaneously enhance its activity, thermostability, and soluble expression yield. The top-performing variant, M8, was rigorously characterized and benchmarked against leading PET hydrolases, and the mechanistic basis for its improved properties was elucidated through structural analysis and molecular dynamics simulations. Finally, we demonstrated the application potential of M8 by achieving long-term depolymerization of high-solid-loading PET in natural seawater. This study provides​ an efficient enzymatic platform for seawater-compatible PET recycling, thereby laying the groundwork for future simultaneous enzymatic depolymerization and fermentation (SEDF) processes.

## Materials and methods

### Materials

The expression vector pET-28a (+)-tac, which contain both the tac and T7 promoters, was used for the mutagenesis library construction and protein expression (Huang et al. 2016). The multiple cloning sites (MCS) and the upstream sequences encoding fusion tags were replaced by a simplified MCS (NdeI-BamHI-HindIII-XhoI). *Escherichia coli* (*E. coli*) DH5α competent cells, *E. coli* BL21 (DE3) competent cells and DNA polymerase were obtained from Takara (Dalian, China). Restriction endonucleases and T4 DNA ligase were sourced from Thermo Fisher Scientific (Hudson, NH, USA). Terephthalic acid (TPA, purity 99%), 5-bromo-4-chloro-3-indolyl acetate (X-C_2_) and 5-bromo-4-chloro-3-indolyl octanoate (X-C_8_) were purchased from Aladdin (Shanghai, China). Mono-(2-hydroxyethyl) terephthalate (MHET, purity 95%) was purchased from Bidepharmatech Ltd. (Shanghai, China). *Para*-nitrophenyl (pNP)-acetate was purchased from Sigma-Aldrich (Sigma-Aldrich, St. Louis, MO). All other chemicals and reagents were of analytical grade and were purchased from commercial sources.

### Gene construction

Codon-optimized nucleotide sequences of BTA1, BTA2, FsC, and LCC, were extracted from a previous study (Tournier et al. 2020). Genes encoding BsEstB (Uniprot: D7R6G8), Cut190 (Uniprot: W0TJ64), *Is*PETase (Uniprot: A0A0K8P6T7), and its variants-including *Is*PETase-M7 (K95N/I168R/P181V/S207E/S214V/N233G/A248D), *Is*PETase-M15 (K95N/T140D/I168R/P181V/S188R/N190D/S207E/N212Q/S214V/N233G/A248D/S278D/T279D/R280E/S290E), *Is*PETase-DuraPETase (L117F/Q119Y/T140D/W159H/G165A/I168R/A180I/S188Q/S214H/R280A/), *Is*PETase-FastPETase (S121E/D186H/R224Q/N233K/R280A), *Is*PETase-HotPETase (S58A/S61V/R90T/K95N/Q119K/S121E/M154G/P181V/Q182M/D186H/S207R/N212K/S213E/S214Y/R224L/N233C/N241C/K252M/T270Q/R280A/S282C), as well as PE-H (Uniprot: A0A1H6AD45), TurboPETase (Cui et al. 2024), CaPETase^M9^ (Hong et al. 2023), Kubu-P^M12^ and Mipa-P^M19^ (Seo et al. 2025) were codon optimized for expression in* E. coli* cells. The DNA sequences were synthesized by GENEWIZ (Suzhou, China). The sequences encoding N-terminal 29 amino acids of *Is*PETase and its mutants were omitted from the synthetic DNA (Han et al. 2017). Signal peptides of Cut190 and PE-H were predicted by SignalP tool (http://www.cbs.dtu.dk/services/SignalP/) and subsequently removed from the synthetic DNA. The genes for *Is*PETase and its mutants were cloned into the BamHI (5’) and XhoI (3’) sites of pET-28a (+)-tac vector. The genes for all the other enzymes were cloned into the NdeI (5’) and XhoI (3’) sites of pET-28a(+)-tac. All expressed proteins contain a C-terminal 6 × histidine tag. The nucleotide and corresponding amino acid sequences are provided in Supplementary data.

### Expression and purification of the proteins

Recombinant proteins were expressed in *E. coli* BL21 (DE3). Cells were cultivated at 37 °C in LB to an optical density at 600 nm (OD_600 nm_) of ~ 0.8. Isopropyl β-D-thiogalactopyranoside (IPTG) was added to a final concentration of 0.6 mM for all proteins except for BsEstB (0.1 mM). The cultures were further incubated at 16 °C with shaking at 180 rpm for 14 h (BsEstB, BTA1, BTA2 and PE-H) or 24 h (FsC, *Is*PETase and its mutants, LCC and LCC-ICCG). For Cut190, the induction condition was 30 °C, 180 rpm and 14 h. Then cells were harvested by centrifugation (6,000 × g, 10 min, 4 °C), resuspended in binding buffer (20 mM Tris–HCl, pH 8.0, 500 mM NaCl, 5 mM imidazole) and disrupted by sonication on ice. After centrifugation (10,000 × g, 20 min, 4 °C), the soluble fractions were loaded onto Ni^2+^-affinity resin (TransGen Biotech, Beijing, China). Unbound proteins were washed with washing buffer (20 mM Tris–HCl, pH 8.0, 500 mM NaCl, 60 mM imidazole), and bound proteins were eluted with elution buffer (20 mM Tris–HCl, pH 8.0, 500 mM NaCl, 1 M imidazole). For enzyme activity analysis, the purified enzymes were dialyzed against Na_2_HPO_4_-HCl buffer (50 mM, pH 7.0) and stored at 4 °C. For crystallization, the target protein was dialyzed against a buffer containing 20 mM HEPES pH 7.0, 50 mM NaCl and 1 mM DTT for two hours. The desalted protein was further purified by heparin-affinity chromatography (GE Healthcare) and eluted with a buffer containing ~ 300 mM NaCl. Fractions containing the target protein at > 90% purity (as assessed by sodium dodecyl sulfate polyacrylamide gel electrophoresis (SDS-PAGE)) were pooled, concentrated to 5 mg/mL with a Millipore centrifugal filter (molecular-weight cutoff 10 kDa), flash frozen in liquid nitrogen and stored at −80 °C. Concentration of the purified protein was determined by Bradford assay (Bradford 1976).

### Preparation of PET powders and crystallinity determination

PET film (250 µm thickness) was purchased from Goodfellow (Shanghai, China, product number ES301445). The film was cut into 5 × 5 mm pieces, submerged in liquid nitrogen for 5 min and then ground. After grinding and sieving, the particle size of the powder was measured by using Mastersizer 2000 (Malvern Instruments Limited, UK). The fraction with D_90_ = 544 ± 7 µm (mean ± s.d., *n* = 3) and D_50_ = 393 ± 4 µm (mean ± s.d., *n* = 3) was used for the determination of PET hydrolyzing activity. The packaging tray of fruits was purchased from a local supermarket (Guangzhou, China). The particle sizes of this post-consumer PET (PC-PET) powder were D_90_ = 450 ± 1 µm (mean ± s.d., *n* = 3) and D_50_ = 207 ± 0.5 µm (mean ± s.d., *n* = 3). The crystallinity of PET powder was analyzed by differential scanning calorimetry instrument DSC-204 F1 (Netzsch, Germany) with a heating rate of 10 °C min^−1^ from 0 to 280 °C. Percentage of crystallinity was calculated based on the equation below:

$$\mathrm{Crystallinity}\;(\%)=\frac{\Delta{\mathrm{H}}_{\mathrm m}-{\Delta\mathrm{H}}_{\mathrm c}}{\Delta{\mathrm{H}}_{\mathrm m}^\circ}\times100$$

∆H_m_ and ∆H_c_ are the enthalpies of melting and cold crystallization, which can be determined by integrating the endothermic melting peak and exothermic cold crystallization peak respectively. ∆H○ m is the melting enthalpy of 100% crystalline polymer, which is ~ 140.1 J/g (Mehta et al. 1978; Vertommen et al. 2005). PET powder sample prepared in our lab has a cold crystallization temperature of 135.4 °C, a melting temperature (T_m_) of 251.5 °C, an enthalpy of cold crystallization (ΔH_c_) of 29.1 J/g, a melting enthalpy (ΔH_m_) of 37.5 J/g and a percentage of crystallinity of 6.0%. PC-PET powder sample has a cold crystallization temperature of 126.1 °C, a T_m_ of 250.9 °C, an enthalpy of cold crystallization (ΔH_c_) of 31.4 J/g, a melting enthalpy (ΔH_m_) of 43.8 J/g and a percentage of crystallinity of 8.9%.

### PET hydrolysis activity assay

PET hydrolysis activities of the enzymes were determined in artificial seawater (pH 8.2, 50 mM Tris–HCl, 24.53 g/L NaCl, 5.20 g/L MgCl_2_, 4.09 g/L Na_2_SO_4_, 1.16 g/L CaCl_2_, 0.695 g/L KCl and 0.201 g/L NaHCO_3_) (ASTM D1141-98. 2021). The purified enzyme (500 nM) was added into 300 μL artificial seawater containing 8 mg PET powder to initiate the reaction. The mixtures were incubated at 30 °C for 18 h. To terminate the reactions, 300 μL methanol was added into the reaction mixture followed by heating at 95 °C for 15 min. After cooling and centrifugation (10,000 × g, 5 min), the samples were filtered using a 0.22 µm filter. The concentrations of released TPA and MHET were determined by high performance liquid chromatography (HPLC). All experiments were performed in three replicates. Since the concentration of bis(2-hydroxyethyl) terephthalate (BHET) in the samples is negligible, the sum of TPA and MHET represents the enzyme activity.

HPLC analysis was conducted on an Alliance HPLC system (Waters) equipped with a SunFire C18 column (5 μm, 4.6 × 250 mm, Waters). The mobile phase was acetonitrile/0.1% (v/v) formic acid in water at a flow rate of 0.8 mL/min. The column temperature was maintained at 30 °C, and the signals were recorded with a UV detector at 260 nm. The elution condition was as follows: 0 to 6 min, 1–5% (v/v) acetonitrile in a linear gradient; 6 to 16 min, 5–70% acetonitrile in a linear gradient; 16 to 22 min, 70–100% acetonitrile in a linear gradient; 22 to 26 min, 100% acetonitrile; 26 to 31 min, 1% acetonitrile. The retention times of TPA and MHET are 15.1 and 15.4 min, respectively. Concentrations in the samples were determined using standard curves prepared with commercial TPA and MHET.

### Construction and screening of saturation mutagenesis libraries

Saturation mutagenesis (SM) libraries targeting individual residues of *Is*PETase were constructed using degenerate NNK codons (N = A/T/C/G, K = G/T) and the TaKaRa MutanBEST Kit. In brief, full-length plasmids were amplified by polymerase chain reaction (PCR). PCR products were blunt-ended, phosphorylated, ligated and then transformed into *E. coli* DH5α competent cells. Library diversity was assessed by the ‘Quick Quality Control’ (QQC) method (Kille et al. 2013). In short, the transformants were cultured on LB agar plates containing 50 μg/mL kanamycin overnight at 37 °C. All colonies from a plate were pooled, and plasmid DNA was isolated. A single sequencing reaction was then performed on the pooled DNA to verify whether all the 32 possible codons were successfully introduced. The primers used were listed in Tab. S1.

For library screening, the transformants were cultured on LB agar plates (50 μM IPTG and 50 μg/mL kanamycin) for 36 h at 30 °C. Plates were incubated for 50 min at 75 °C. After cooling to room temperature, 10–15 mL of a reaction mixture-containing 25 mM Na_2_HPO_4_-HCl (pH 7.0), 0.125 mM X-C_2_ and 0.5% (w/v) molten agar was poured onto the plates. Then the double-layered plates were incubated at 30°C for 3 h. Blue colonies were considered as positive mutants. Their plasmids were extracted and transformed into competent *E. coli* BL21 (DE3). The mutations were identified by DNA sequencing. To ensure 98% library coverage, more than 160 clones were screened for each NNK library (Reetz et al. 2008).

The mutants of LCC, *Is*PETase-M6 and *Is*PETase-M7 were constructed by site-directed mutagenesis according to the molecular manipulation described above. The correctness of all the mutants was confirmed by DNA sequencing. The primers used were listed in Tab. S1.

### Thermostability analysis

The thermostability of *Is*PETase and its mutants was evaluated by determining T_50_ and melting temperature (T_m_)_._ T_50_ is defined as the temperature where 50% of the protein is inactivated in 10 min. In details, the samples were heated at different temperatures (typically 35–80 °C) for 10 min. After heat treatment, the residual activity was measured. Enzymes were incubated with 2 mM pNP-acetate in 50 mM Na_2_HPO_4_-HCl (pH 7.0) and 10% (v/v) methanol at 40 °C for 5 min. The released pNP was monitored at 405 nm to determined its concentration. The T_50_ values were determined by fitting a shifted sigmoid function to the inactivation curves (Romero et al. 2015).

T_m_ values were determined by a fluorescence-based thermal stability assay. Purified protein (5 μg) was mixed with Protein Thermal Shift Buffer (5 μL) and Protein Thermal Shift Dye (8 ×, 2.5 μL) (Thermo Fisher Scientific, Hudson, NH, USA) in a final volume was 20 μL using 8-strip PCR tubes (Monad, Shanghai, China). The mixture was heated from 25 to 99 °C in a StepOnePlus real-time PCR system (Applied Biosystems, Foster City, CA, USA). The T_m_ values were determined from the peaks of the first derivatives of the melting curve using the StepOne software.

### Soluble expression analysis

Soluble expression levels of the enzymes were analyzed by SDS-PAGE and western blot (WB). After induction, 1 mL culture was collected and centrifuged (10,000 × g, 10 min). The cells were resuspended in 1 mL Na_2_HPO_4_-HCl buffer (50 mM, pH 7.0) and disrupted by sonication on ice. A 200 µL aliquot of the total lysate was saved as the whole-cell sample. The remaining lysate was centrifuged (10,000 × g, 10 min, 4 °C) to separate the soluble fraction (supernatant) from the insoluble fraction (pellet). The pellets were resuspended in 800 µL of the same buffer. Samples (40 μL) were mixed with loading buffer (10 μL, 250 mM Tris–HCl, pH 6.8; 10%, SDS, w/v; 0.5% bromophenol blue, w/v; 50%, glycerin, v/v; 5% β-mercaptoethanol, v/v), heated at 98 °C for 10 min, and applied to 12% SDS-PAGE. The loading volume for each sample was 20 μL. After electrophoresis, the gels were stained with Coomassie brilliant blue R250.

For WB analysis, the loading volume was 7.5 μL per lane. The samples of *Is*PETase and its single point mutants were prepared according to the description above. The supernatants of mutants M8, P181A, P181V, DuraPETase and LCC-ICCG were diluted at different ratios and then mixed with loading buffer. Samples were separated on 12% SDS-PAGE and then electro-transferred to poly (vinylidene fluoride) (PVDF) membrane in ice bath. The membrane was blocked by TBST solution (20 mM Tris–HCl, pH 7.5, 500 mM sodium chloride, and 0.05% Tween-20, w/v) containing 5% (w/v) skim milk for 1 h, and incubated for 2 h at room temperature with a mouse anti-His-tag antibody (Clone No 1B7G5, 1500 μg/mL, 1: 5000 dilution, Proteintech, Chicago, USA). Subsequently, it was washed five times by TBST solution, incubated for 0.5 h at room temperature with horseradish peroxidase (HRP)-conjugated goat anti-mouse antibody (200 μg/mL, 1: 5000 dilution, Proteintech) and washed additional four times. Chemiluminescence detection was carried out with Immobilon Western Chemiluminescent HRP Substrate (Millipore Corporation, Billerica, USA). The chemiluminescent signals were captured by Tanon5200 system (Tanon, Shanghai, China). Grayscale analysis of the chemiluminescent bands was performed by using ImageJ.

### Crystallization, data collection and structure determination

Crystallization was performed using the sitting drop vapor diffusion method at 25 °C. The sample and the well solution were mixed at a 1:1 volume ratio. Crystals were obtained with the reservoir condition of 1.8 M (NH_4_)_2_SO_4_, 0.1 M NaCl and 0.1 M MES (pH6.0). The fully-grown crystals were transferred to a cryo-protectant solution (10% (v/v) glycerol plus the reservoir solution) and were flash frozen in liquid nitrogen.

The X-ray diffraction data were collected using beamline 19U1 (BL19U1) at the Shanghai Synchrotron Radiation Facility (SSRF, Shanghai, P.R.China) (Zhang et al. 2019). The data were processed using *HKL3000* (Otwinowski and Minor 1997) and initial phasing of M8 structure was carried out by molecular replacement using the *Is*PETase structure (PDB 6EQE) (Austin et al. 2018) as the search model with phenix (McCoy et al. 2007). The model-building and refinement were carried out by Coot (Emsley et al. 2010) and Phenix.refinement (Adams et al. 2010) respectively. TLS refinement was employed, generating a final model with the R_free_ and R_work_ values of 0.179 and 0.155, respectively. The model has 98.08, 1.92, and 0% of the residues falling into the favored, allowed, and disallowed regions in the Ramachandran plot. The structural figures were produced with PyMOL (www.pymol.org). The final model was validated by Molprobity (Chen et al. 2010). All the data collection and refinement statistics were presented in Tab. S2.

### Molecular docking and molecular dynamics simulations

The structure of *Is*PETase was derived from the PDB database (PDB: 6EQE). The structure of the variant M8 was solved by X-ray crystallography in this work (PDB 7VWN). The ligand molecule, PET pentamer: 2-hydroxyethyl-(monohydroxyethyl terephthalate)_5_ (2-HE(MHET)_5_) was constructed and optimized using the software Avogadro 1.2.0 (Hanwell et al. 2012). Semi-flexible docking was carried out by using AutoDock Vina (version 1.1.2) (Trott and Olson 2010). The center coordinates of grid box were: x: −18.475, y: −1.62, z: 9.082, and the search space size was 30 × 40 × 30 Å^3^. The best docking model was selected according to the following rules: (1) the substrate 2-HE(MHET)_5_ was bonded to the subsite I, subsite IIa, IIb and IIc of the enzyme; (2) the distance between the carbonyl carbon of 2-HE(MHET)_5_ and the hydroxyl oxygen atom of S160 was less than 4 Å; (3) the distance between the carbonyl oxygen of 2-HE(MHET)_5_ and the oxyanion hole was proper; (4) the model that met the above conditions and showed the lowest docking energy was selected as the final model.

All the molecular dynamics (MD) simulations were performed with GROMACS 2019.3 (Abraham et al. 2015) using the AMBER14SB force field (Maier et al. 2015) for protein. H + + (Gordon et al. 2005) was used to estimate the protonation state of the titratable residue at pH 8.2, and the δ nitrogen atom of the catalytic residue H237 (HID) was protonated according to the catalytic mechanism of esterase. The force constant parameters of 2-HE(MHET)_5_ molecule were generated by Antechamber module (Wang et al. 2006) along with the General AMBER force field (Wang et al. 2004). The RESP2(0.5) (Schauperl et al. 2020) atomic partial charges for 2-HE(MHET)_5_ was calculated at the B3LYP/def2TZVP level with Gaussian 09 software (Frisch et al. 2013). Furthermore, the critical post-processing steps in this Gaussian calculation workflow were implemented via Multiwfn (Lu 2024; Lu and Chen 2012). All systems were solvated in a cubic box with a distance of 10 Å between the edge of the box and the surface of the protein, and were filled with TIP3P water molecules. To better simulate the experimental conditions, ions corresponding to the artificial ocean water (109 Na^+^, 12 Mg^2+^, 2 Ca^2+^, 2 K^+^ and 6 SO_4_^2−^) were added into the box. Then 132 chloride ions were added to neutralize the system. The total concentration of ions was 0.5 M.

The systems were minimized with 2000 steps of steepest descent minimization and 3000 steps of conjugate gradient minimization. Then restricted equilibrium simulations (1000 kJ/(mol nm^2^) for the solute heavy atoms) were performed for each system with 200 ps NVT simulation (heating the system temperature up to 303.15 K) and 500 ps NPT simulation (adjusting the system pressure to 1 bar). Temperature and pressure were controlled using the modified V-rescale thermostat (Bussi et al. 2007) and the Parrinello-Rahman barostat (Parrinello and Rahman 1981) with damping coefficients of 0.1 and 2 ps, respectively. For free *Is*PETase and M8, a 200 ns of unrestrained MD simulation was performed. For 2-HE(MHET)_5_ enzyme complexes, the best docking models were selected as the initial structures. To increase the conformational stability of the complex, MD simulations of 200 ns were performed using a restraint force of 1000 kJ/(mol nm^2^) on the Cartesian coordinates of 2-HE(MHET)_5_ obtained from molecular docking. The time step was 2 fs. Periodic boundary conditions were applied and the LINCS algorithm (Hess et al. 1997) was used to constrain the hydrogen atoms. The long-distance electrostatic interaction was calculated by particle-mesh Ewald (PME) method (Darden et al. 1993) and short-range interactions were cut off at 10 Å.

The binding free energy (ΔG_bind_) between the substrate and the protein was calculated by using molecular mechanics/generalized Bohr surface area (MM/GBSA) method with gmx_MMPBSA1.4.3 software (Valdes-Tresanco et al. 2021). The entropy change of the system was calculated using the Interaction Entropy approximation (IE) method (Duan et al. 2016). After 200 ps NVT simulation and 500 ps NPT simulation, a short time of 4 ns unrestrained MD simulation was performed. ΔG_bind_ was computed from 1000 frames. For each system, simulation was repeated three times and the average ΔG_bind_ was obtained. The temperature for entropy calculation was set as 303.15 K. The implicit Generalized Born (GB) solvent model (GB^OBC^, igb = 5) (Onufriev et al. 2004) was used with the salt concentration set to 0.5 M.

### Enzymatic PET depolymerization in natural seawater

Depolymerization reactions were performed in 5 mL of natural seawater (collected from the South China Sea) using PC-PET powder as substrate. Key reaction parameters, including substrate loading (5–15%, w/v) and enzyme concentration (500–2000 nM), were investigated. All reactions were carried out at either 30 °C or 37 °C. ​​To counteract acidification from hydrolytic products, the reaction pH was actively maintained at 8.2 by adding NaOH solution at 6-h intervals, notwithstanding the presence of 50 mM Tris–HCl as a buffering agent.​ To avoid contamination, all buffer and enzyme solutions were filter-sterilized (0.22 µm), and the Erlenmeyer flasks fitted with glass stopper were sterilized and dried prior use. At designated time points, samples were withdrawn, mixed with an equal volume of methanol, and heated at 95 °C for 15 min to terminate the reaction. After cooling, samples were centrifuged and filtered (0.22 µm), and the concentrations of TPA and MHET were quantified by HPLC.

## Results

### Selecting highly-active PET hydrolase in artificial seawater

**Fig. 1:**
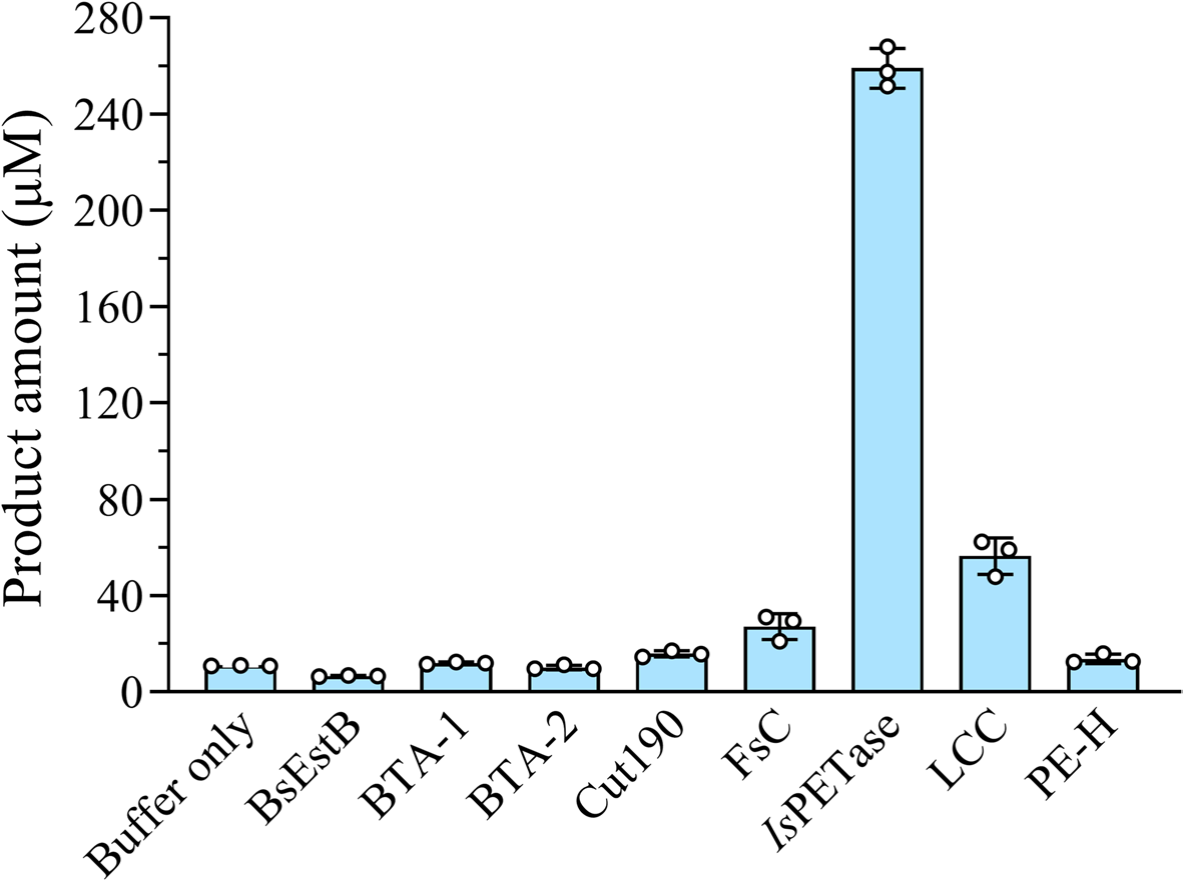
The activity of PET-hydrolases towards PET powder in artificial seawater. The reaction mixtures consist of 500 nM purified proteins, 8 mg PET powder (crystallinity of 6%) and 300 μL artificial seawater. All the assays were carried out at 30 °C for 18 h. The bars and cycles are the average and individual numbers of triplicate measurements, respectively. The error bars represent standard deviation

We first performed phylogenetic analyses on the potential enzymes for PET hydrolysis and identified several candidates including the esterases from *Bacillus subtilis* (BsEstB) (Ribitsch et al. 2011), *Thermobifida fusca* (BTA1 and BTA2) (Then et al. 2015), *Pseudomonas aestusnigri* (PE-H) (Bollinger et al. 2020), the polyesterase from *Saccharomonospora viridis* (Cut190) (Kawai et al. 2014), the cutinases from *Fusarium solani pisi* (FsC) (Vertommen et al. 2005), the leaf-branch compost cutinase (LCC) from a metagenomic library (Sulaiman et al. 2012) and the PETase from *Ideonella sakaiensis* (*Is*PETase) (Yoshida et al. 2016) (Fig. S1). These enzymes were expressed and purified to homogeneity, and were assayed for their ability to hydrolyze PET powder (crystallinity of 6.0%) at 30 ℃ in an artificial seawater prepared according to ASTM D1141-98. Among the 8 enzymes tested, *Is*PETase demonstrated the highest activity, outperforming the second runner-up by > 4.5 folds (Fig. 1). However, the thermostability of *Is*PETase is poor with a half-life of ~ 1 d at 30 ℃ (Huang et al. 2018). Hence, we seek to improve its thermostability by protein engineering.

### Screening for thermostable *Is*PETase variants

**Fig. 2:**
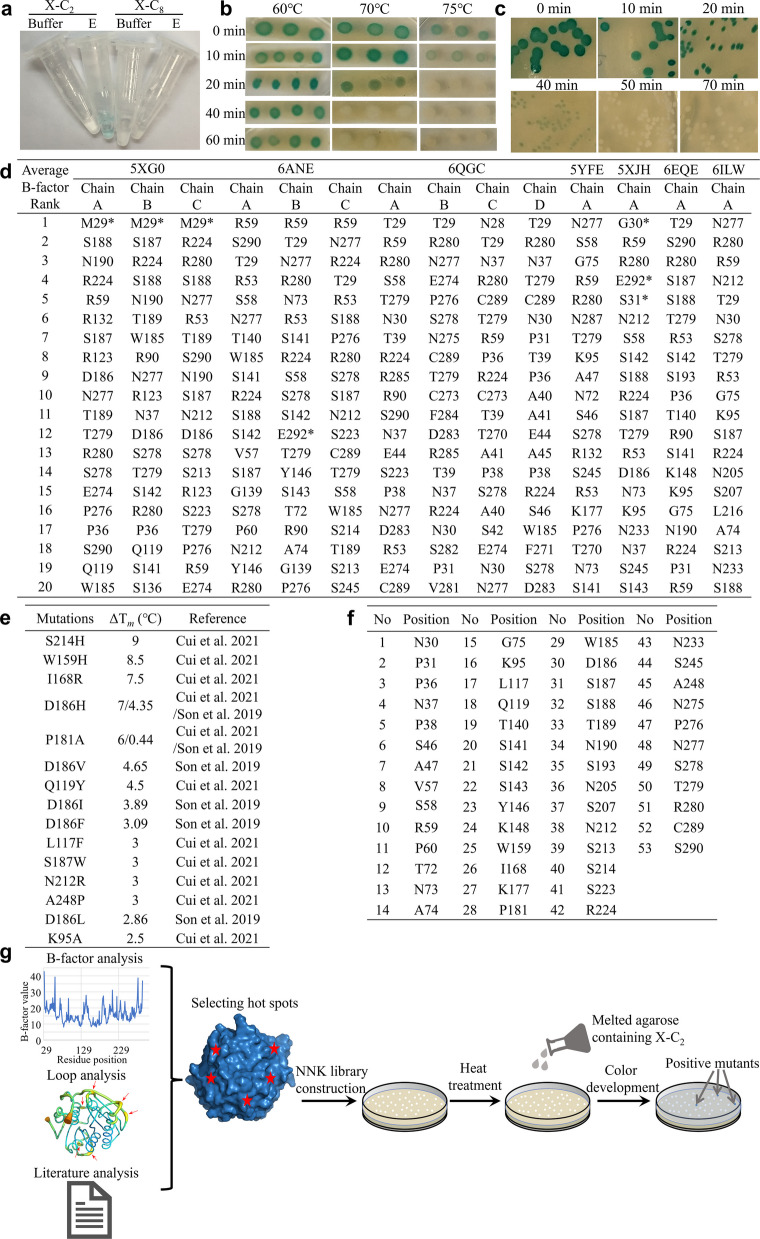
Engineering of *Is*PETase for thermostability enhancement. **a**-**c** The modified Petri dish-based double-layer screening method for thermostable *Is*PETase mutants. **a** The activity test of *Is*PETase towards 5-bromo-4-chloro-3-indolyl acetate (X-C_2_) and 5-bromo-4-chloro-3-indolyl octanoate (X-C_8_). Buffer: 50 mM Na_2_HPO_4_-HCl (pH 7.0). Enzyme: addition of 0.1 μg purified *Is*PETase. **b** The product visualization (indicated by the blue colonies) over a time course of 1 h under different temperatures. The colonies were formed by the host *Escherichia coli* DH5α carrying an empty vector. **c** The *Is*PETase activity over a 70-min period at 75 °C. The colonies were formed by DH5α carrying the encoding gene of *Is*PETase. **d**-**f** Selection of the specific candidate residues for construction of the saturation mutagenesis (SM) libraries. **d** The list of the specific candidate residues and their PDB sources. The top 20 residues from 14 chains of 7 reported crystal structures of *Is*PETase were ranked by B-factors. The residues marked by * were introduced for recombinant expression, so they were not selected. **e** The 11 extra positions added to the list where beneficial mutations substantially increased melting temperatures (T_m_) values based on previous studies. **f** The comprehensive list of the final 53 amino acid positions used for the SM studies. **g** The flow chart of the engineering strategy used in this study B-factor analysis was performed by using B-FITTER (Reetz and Carballeira 2007). Loop analyis was conducted according to Yu and Huang (2014). Hot spots refer to theamino acids selected for mutagenesis (details see Section 3.2 of the Results). Red arrows indicate loop regions, and red asterisks indicate hot spots

To efficiently screen for mutants with improved thermostability, we modified a Petri-dish-based double-layer functional screening method (Huang et al. 2016) using the promiscuous activity of *Is*PETase towards the chromogenic substrate 5-bromo-4-chloro-3-indolyl acetate (X-C_2_) (Fig. 2a). Upon heat treatment, the *E. coli* DH5a strain harboring the *Is*PETase gene would lyse, releasing the enzyme, which then converts X-C_2_ in the top agar layer to a blue product (Fig. 2b, c, g). The esterase of DH5a itself was inactivated by an incubation of 20 min at 75 ℃ (Fig. 2b), and *Is*PETase lost activity under a harsher condition (50 min at 75 ℃) (Fig. 2c). By visually checking the blue colonies on the plates after the heat treatment, positive mutants stood out (Fig. 2g).

**Fig. 3:**
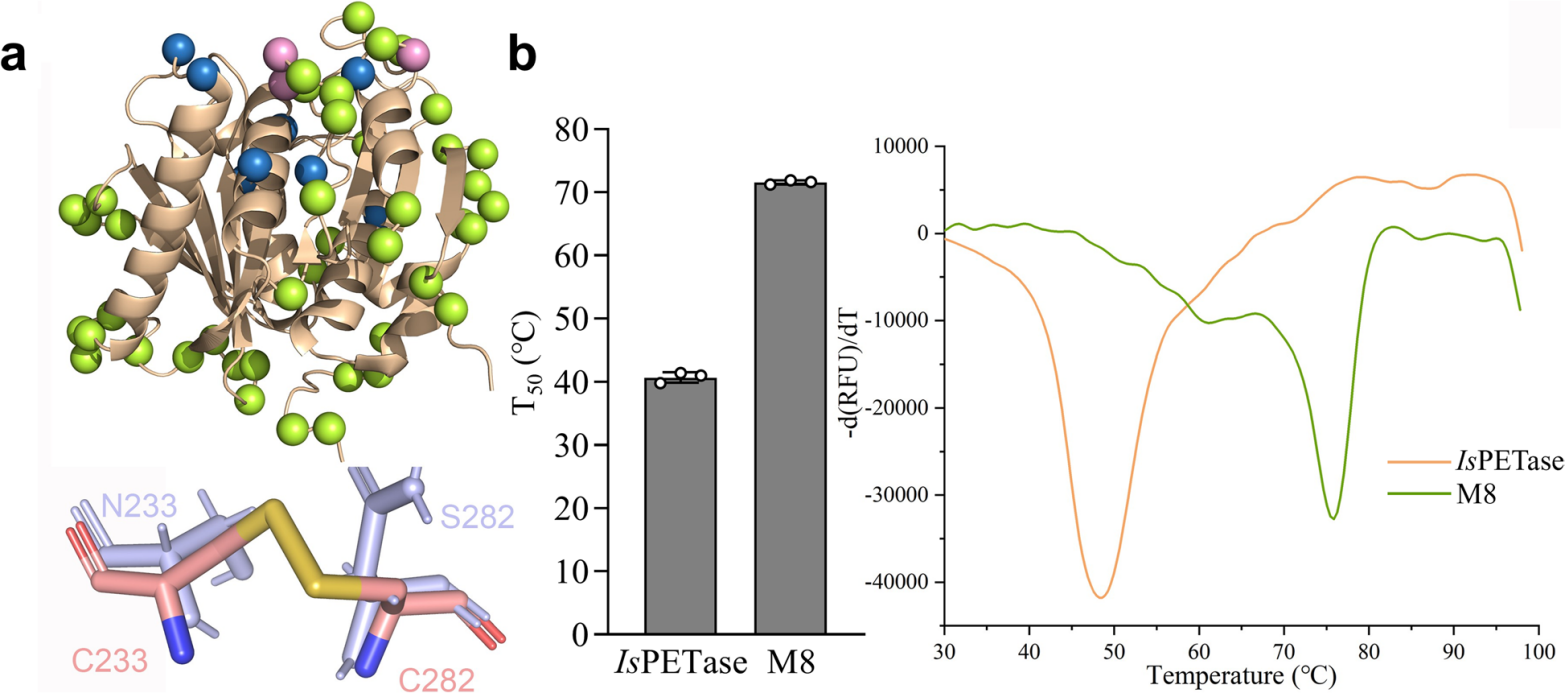
Improvement of *Is*PETase thermostability by saturation mutagenesis and the introduction of a disulfide bridge. **a** Locations of the 53 resides for saturation mutagenesis (up) and the disulfide bridge introduced (bottom). The protein was shown as wheat cartoon (PDB 6EQE). The Cα of the residues was shown as limon sphere (selected by B-factor analysis and loop analysis), blue sphere (selected by literature analysis), or pink sphere (three residues that were overlapped). The original residues N233 and S282 in *Is*PETase were shown as light blue sticks, and that formed disulfide bridge in M8 (C233 and C282) were showed as salmon sticks **b** T_50_ values (left) and melting temperatures (T_m_) (right) of *Is*PETase and its mutant M8. First derivatives were calculated from thermal denaturation curves and the peaks correspond to protein T_m_. Each curve is a representative of a triplicate test

To identify target residues for mutagenesis, we analyzed flexible sites in available *Is*PETase structures, hypothesizing that rigidifying these regions would enhance thermostability while minimizing structural disruption (Yu and Huang 2014). Then 45 such residues with the highest B-factors (the top 20) from the 14 chains of the 7 *Is*PETase structures (Protein Data Bank (PDB; https://www.rcsb.org) ID: 5XG0, 6ANE, 5YFE, 5XJH, 6EQE, 6ILW, and 6QGC) were selected by using the program B-FITTER (Reetz and Carballeira 2007) (Fig. 2d). Meanwhile, 11 positions where beneficial mutations increased the melting temperature (T_m_) by 2 ℃ or higher (Cui et al. 2021; Son et al. 2019) were also added to the mutagenesis list (Fig. 2e). Three sites (D186, S187 and N212) fall into both categories and the final combination produced a total of 53 positions (Figs. 2f, 3a). Site saturation mutagenesis (SM) libraries were constructed at these designated sites to introduce the 32 possible codons by using degenerate codon NNK (N = A/T/C/G, K = G/T), which encoded all possible 20 amino acids. The libraries were subsequently screened by the method above and 22 positive mutants coming from 17 positions substantially increased the thermostability of *Is*PETase. Compared to wild-type (WT), the T_50_ values of these mutants increased 0.8–9.8 ℃, respectively, with the highest being the P181V (Fig. S2a).

### Combinational mutagenesis

We next considered the possibilities of combining multiple mutations for higher improvement on the thermostability of *Is*PETase, and meanwhile tried to maintain its high activity at 30 ℃. Since several mutations occurred on the same residue (K95, P181, N233 and T279) more than once (Fig. S2a), they were preferentially selected for the combinations. Additionally, D186H and I168R would conflict with each other^68^, so we kept the latter, which brought higher stabilizing effects (Fig. S2a). W159H would probably reduce the hydrolysis activity of the enzyme^68^, and was removed from the list. Consequently, the remaining mutations were K95N, T140D, I168R, P181V, S188R, N190D, S207E, N212Q, S214V, N233G, A248D, S278D, T279D, R280E and S290E, respectively, seven of which each increased the T_50_ values by more than 3 ℃ (Fig. S2a). Therefore, two multi-mutants were created: M7 (K95N/I168R/P181V/S207E/S214V/N233G/A248D) and M15 (containing all the aforementioned mutations).

The T_50_ values of M7 and M15 were 65.6 and 62.4 °C, respectively, representing increases of 24.9 and 21.7 °C over the WT (Fig. S2b). However, their activities were only 29% and 25% of that of WT (Fig. S2c). Hence, we resumed our efforts on M7 to restore the hydrolysis activity. Structural analysis suggested that the S207E mutation, while increasing T₅₀ by 4.6 °C (Fig. S2a), might impair activity due to its location within the active site adjacent to the catalytic residue D206. Therefore, we eliminated this mutation to give rise to M6 (K95N/I168R/P181V/S214V/N233G/A248D) and restored the activity to 92% of the WT level (Fig. S2c) with a decrease of 2.1 °C in T_50_ (Fig. S2b). We further tested all the other 15 single mutations in combination of M6 for higher thermostability enhancement, and found that only M6-W159H and M6-S278D greatly improved thermostability but with impaired activities (Fig. S2d, e). Then the R280A mutation with reported beneficial effects (Joo et al. 2018) was introduced, boosting the activity to 117% of the WT level (*P* = 0.0041, one-sided t test) with only a slight decrease of 0.8 °C in T_50_ (Fig. S3c, d). Moreover, a disulfide bond (G233C-S282C) was introduced into M6-R280A based on prior results of Cut190 from *Saccharomonospora viridis* (Oda et al. 2018), TfCut2 from *Thermobifida fusca* KW3 (Then et al. 2016), LCC (Tournier et al. 2020) and a mutant of *Is*PETase named TS-PETase (Zhong-Johnson et al. 2021) (Fig. 3a, Fig. S3a, b), greatly improving the T_50_ by 8.9 °C without comprising the activity (Fig. S3 c, d). The T_50_ and T_m_ values of the optimal mutant M8 (M6-R280A-G233C-S282C) were 71.6 and 75.8 °C respectively (30.9 and 27.3 °C higher than that of WT) (Fig. 3b), while its hydrolysis activity 114% of WT (294.2 μM vs 259.0 μM,* P* = 0.0034, one-sided t test).

**Fig. 4:**
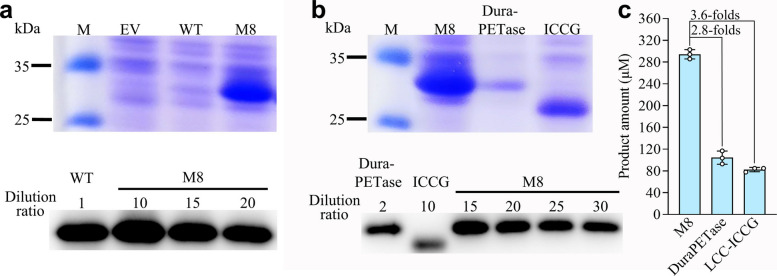
Improved soluble expression of M8 and property comparation with DuraPETase and LCC-ICCG. **a** The soluble expression of *Is*PETase and M8. **b** The soluble expression of M8, DuraPETase and LCC-ICCG. Gray analyses of the bands in western blots (three biological replicates) showed that the soluble expression yield of M8 was 14.3-, 11.5- and 2.9-folds that of *Is*PETase, DuraPETase and LCC-ICCG, respectively. Abbreviations: kDa, kilodaltons; M: marker; EV, empty vector; WT, wild-type *Is*PETase. The proteins were induced by 0.6 mM isopropyl β-D-thiogalactopyranoside (IPTG) at 16 °C for 24 h. See methods for details about experimental protocol. **c** The hydrolysis activity. The reactions were performed at 30 °C for 18 h in artificial seawater with 500 nM purified proteins. The bars and cycles are the average and individual numbers of triplicate measurements, respectively. The error bars represent standard deviation. Uncropped gels and blots are provided in Fig. S6

In addition to hydrolysis activity and thermostability, the soluble expression level is another factor that determines the usability of PET hydrolase in practical application. Interestingly, the soluble expression yield of M8 was 14.3-folds that of the WT (Fig. 4a) while their growth profiles were similar (Fig. S4a). So, we checked M6, M7 (Fig. S4a, b) and all the 22 single point mutants (Fig. S4c, d, e), and established that the mutation at position 181 contributed to the higher soluble expression (Fig. S4d, f). Consistently, introducing the V181P reversion into M7 reduced its soluble expression to the WT level (Fig. S4b). Further characterization confirmed that the P181A and P181V mutations, introduced into the WT *Is*PETase background, significantly increased protein solubility (Fig. S4f). This enhancement is likely attributable to the formation of a more extensive β-sheet structure (Son et al. 2019), which would facilitate the folding of the protein.

### Property comparison with other PET degrading enzymes

The properties of M8 were further benchmarked against two engineered, high-performance PET hydrolases: DuraPETase (an *Is*PETase variant, Cui et al. 2021) and LCC-ICCG (an LCC variant, Tournier et al. 2020). The soluble expression yield of M8 was 11.5- and 2.9-folds that of DuraPETase and ICCG (Fig. 4b, Fig. S5), while hydrolysis activity of M8 was 2.8- and 3.6-folds (Fig. 4c). These results indicated that the total catalytic efficiency (yield × activity) of M8 would be 32.2- and 10.4-folds of that of DuraPETase and ICCG for a single batch under the same cultivation condition. Furthermore, the overexpression of DuraPETase and ICCG inhibited the growth of host cells (Fig. S5), a factor that could limit high-cell-density fermentation and increase enzyme production costs.

### Mechanisms for the property enhancement

**Fig. 5:**
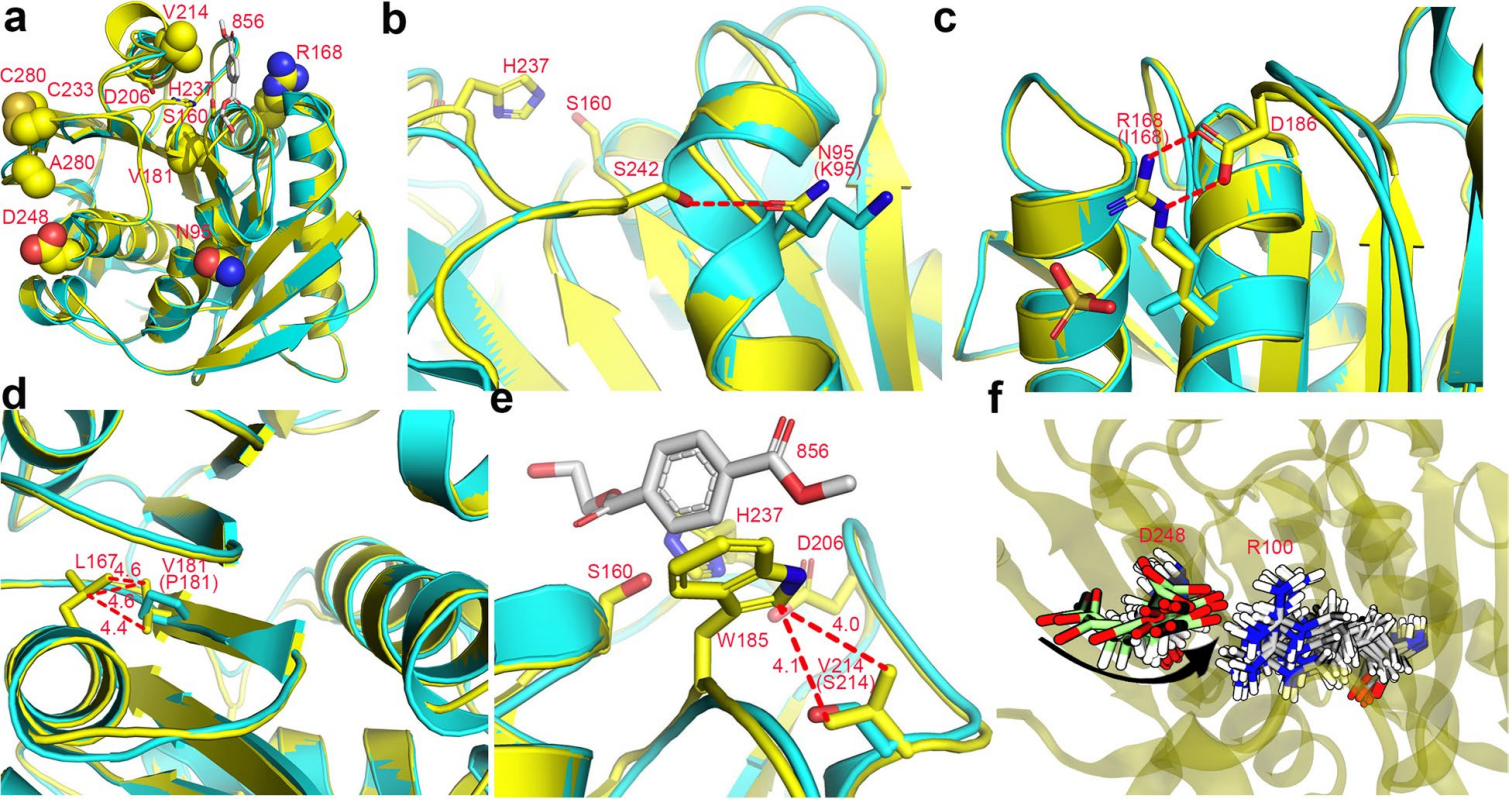
The structural explanation for thermostability enhancement of M8. **a** The overall structure of M8 and distribution of the mutation sites. M8 (yellow, PDB 7VWN) was superimposed onto that of wild-type (WT) *Is*PETase (cyan, PDB 6EQE). The catalytic triad of WT were shown in sticks, and the residues after mutations were shown in spheres. The substrate 856 (O 4-(2-hydroxyethyl) O 1-methyl benzene-1,4-dicarboxylate) was introduced by superimposition as well (silver), with the coordinates from PDB 5XH3. **b**-**e** The local environments of each mutation. The interaction patterns were shown for K95N (**b**), I168R (**c**), P181V (**d**) and S214V (**e**). The interactions were shown by the red lines and the distances of the non-hydrogen bonding interactions were indicated. **f** In the structure of M8, D248 and R100 had no interactions (distance of 6.7 Å), whereas molecular dynamic simulations (Fig. 6a) showed that R100 can rotate to form a salt bridge with D248. The rotation of D248 from the initial conformation was marked by an arrow. The occurrence of the salt bridge was 79.8% (2000 frames, cutoff distance = 6.0 Å)

**Fig. 6:**
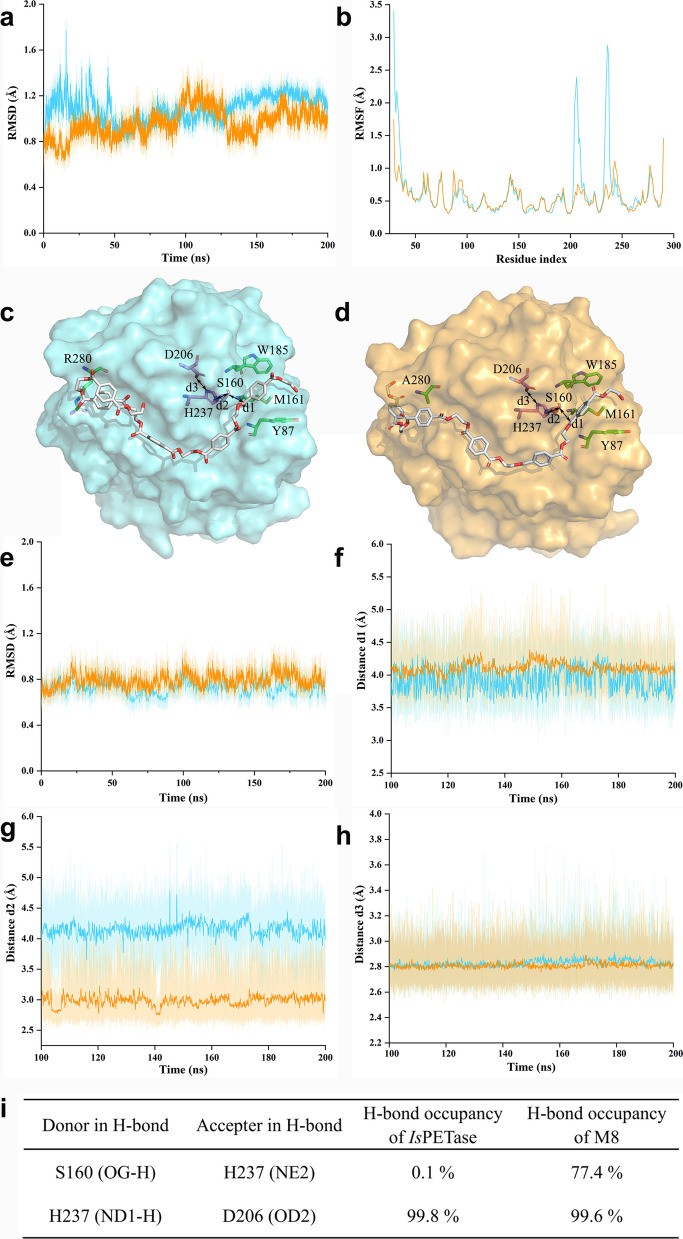
Molecular docking and molecular dynamic simulations on *Is*PETase (blue) and mutant M8 (orange). **a**-**b** Comparison of protein backbone flexibility using root mean square deviation (RMSD) (**a**) and root mean square fluctuations (RMSF) (**b**) of Cα atoms along molecular dynamic (MD) simulations of enzymes in the free state. The flexibility of loop regions 202–218 and 230–240 was much lower in M8 than that in *Is*PETase (**b**), which may confer stability of the enzyme. The best docking models of 2-HE(MHET)_5_ in *Is*PETase (**c**) and M8 (**d**) showed similar conformation except for the MHET around position 280. The spatial clash between R280 and the last benzene ring of 2-HE(MHET)_5_ in *Is*PETase forced the substrate to turn back (**c**). Replace arginine (R) with a small alanine (A) eliminated the clash (**d**), which may facilitate the substrate binding. The catalytic residues S160, D206, H237 (purple), oxyanion hole Y87, M161 (green) and key residue W185 (green) were also shown. Meanwhile, three distances that are critical to catalysis were marked by double-headed arrows. They are: d1, the distance between substrate cleavage site and hydroxyl oxygen of S160; d2, the distance between hydroxyl oxygen of S160 and NE2 of H237; d3, the distance between ND1 of H237 and carbonyl oxygen of D206. During the last 100 ns of MD simulations of enzymes in complex with 2-HE(MHET)_5_ (**e**), d1 and d3 in *Is*PETase and M8 are similar (**f**: average d1 is 3.9 and 4.1 Å respectively; **h**: average d3 is 2.8 and 2.8 Å respectively), whereas d2 in M8 is much shorter (**g**: average d2 is 4.2 and 3.0 Å respectively). Accordingly, the occurrence of H-bond in the M8 simulation between hydroxyl oxygen of S160 and NE of H237 was higher than that of *Is*PETase (**i**), which may be beneficial to the proton transfer between them, thereby improving the catalytic ability of M8

To understand the structural basis of the property enhancement, we solved the crystal structure of M8 (Tab. S2). The overall fold of *Is*PETase was preserved and M8 could be superimposed to that of WT with the root mean square deviation (RMSD) value of 0.178 Å (Fig. 5a). K95N changes the long flexible side chain of the lysine residue to a shorter asparagine, which forms a hydrogen bond with S242 (Fig. 5b). The I168R converted a hydrophobic residue to a charged residue with a long side chain, which forms a salt bridge with D186 (Fig. 5c). P181V enhances the hydrophobic interaction between this residue and L167 (Fig. 5d). S214 is close to the substrate-binding site, and its mutation to a valine places it in a hydrophobic contact distance range (4.0 Å) to W185 (Fig. 5e). While it is difficult to predict the consequences of the A248D based only on the static structure, our molecular dynamics (MD) simulations demonstrated that it can form a salt bridge with the rotated R100 (Figs. 5f, 6a). Consistent with the above results, the rigidity of the loop regions 202–218 and 231–240 was also remarkably increased (Fig. 6b).

The boosted PET-hydrolyzing activity of M8 was mainly due to the mutation R280A (Fig. S3d), which may reduce the spatial conflicts between R280 and the last phenyl ring of the substrate 2-HE(MHET)_5_ (Fig. 6c, d) and thus promotes its binding. Accordingly, molecular mechanics/generalized Bohr surface area (MM/GBSA) calculations also predicted a higher affinity of M8 towards 2-HE(MHET)_5_ (ΔΔG = −2.49 kcal mol^−1^). Furthermore, MD simulations of the enzymes in complex with 2-HE(MHET)_5_ revealed that the average distance between catalytic residues S160 and H237 in M8 was dramatically shorter than that in WT (Fig. 6e-i), implying an easier way for proton shuttling during catalysis.

### Efficient depolymerization of post-consume PET by *Is*PETase-M8 in natural seawater

**Fig. 7:**
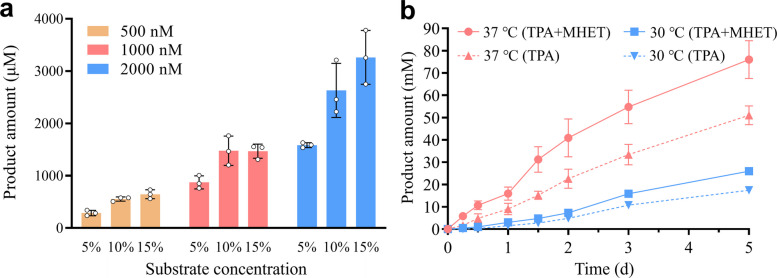
Depolymerization of post-consume PET by *Is*PETase-M8 in seawater. **a** Effects of substrate loading (5–15%, w/v) and enzyme dosage (500–2000 nM) on product yield. The reactions were carried out at 30 °C for 18 h. **b** Time-course monitoring of TPA accumulation and total soluble products (TPA + MHET) at 30 and 37 °C. The reactions were carried out with an enzyme concentration of 2000 nM and a PET loading of 15% (w/v). Error bars represent standard deviation from triplicate experiments. Data points are the average of triplicate measurements, where error bars represent standard deviation

To evaluate the practical potential of *Is*PETase-M8 for industrial PET recycling under saline conditions, we evaluated its depolymerization performance on post-consumer PET in natural seawater. Key parameters, including substrate loadings (5–15%, w/v) and enzyme concentrations (500–2000 nM), were tested at 30 °C over 18 h. As shown in Fig. 7a, the product yield increased with both substrate loading and enzyme concentration, reaching a maximum of approximately 3.3 mM at the highest level of both parameters.

We then monitored extended time-course depolymerization at 30 and 37 °C over 5 days (Fig. 7b). Throughout the testing period, product release increased in an approximately linear manner at both temperatures.​​ At 37 °C, the total soluble products (TPA + MHET) reached 76.8 mM, of which 51.2 mM (66.7%) was TPA. At 30 °C, the total products were 25.9 mM, with 17.4 mM as TPA (67.2%). The average monomer production rate was calculated to be ​​15.4 mM/d​​ at 37 °C and ​​5.2 mM/day​​ at 30 °C. The efficient and sustaineddepolymerization, unimpeded by the complex composition of natural seawater, underscores the strong potential of *Is*PETase-M8 for low-temperature PET recycling ​​in saline environments​​.

## Discussion

In recent years, significant research efforts have been devoted to enzymatic depolymerization of PET, leading to remarkable progress. Under high-temperature and freshwater conditions, high-loading substrates (15–20%, w/v) can now be degraded within 10 h (Cui et al. 2024; Zheng et al. 2024). Concurrently, microbial strains and engineered chassis capable of assimilating the monomers TPA and EG have been developed, achieving uptake rates of 3–28.5 mM/day (Narancic et al. 2021; Werner et al. 2021). However, this conventional stepwise process faces inherent challenges: the high energy cost of maintaining elevated temperatures for enzymatic hydrolysis, the necessity for subsequent cooling to accommodate mesophilic fermentation, and substantial freshwater consumption, which imposes both economic and environmental burdens (Wei et al. 2025). Guided by the principles of next-generation industrial biotechnology (NGIB), we propose a ​​simultaneous enzymatic depolymerization and fermentation (SEDF) process operating directly in seawater at ambient temperatures​​. This integrated approach ​​mirrors the well-established simultaneous saccharification and fermentation (SSF) framework​​ in lignocellulosic bioconversion (Liu et al. 2019), but transcends its limitations by eliminating the need for freshwater. Performing the entire process under saline and mesophilic conditions represents a paradigm shift that could drastically reduce energy input and operational complexity, offering a more sustainable route for PET bio-recycling.

A key prerequisite for implementing SEDF is the availability of an efficient PET-depolymerizing enzyme adapted to saline and low-temperature environments. In this study, we initially screened 8 representative PET hydrolases at 30 °C in artificial seawater. Notably, *Is*PETase, renowned for its high low-temperature activity, outperformed all other enzymes—including thermostable hydrolases such as Cut190 and LCC, which originate from thermophilic microorganisms and compost (Fig. 1). Through iterative protein engineering, we developed the variant *Is*PETase-M8, which exhibits concurrent improvements in thermostability (Fig. 3), catalytic activity (Fig. S3), and soluble expression yield (Fig. 4). This combination of enhanced properties enabled M8 to achieve a monomer production rate of 15.4 mM/day from PC-PET in natural seawater (Fig. 7). Simultaneous optimization of multiple enzyme properties through combinatorial mutagenesis is an attractive but ambitious goal, and a long-standing challenge in protein engineering, due to the complex epistasis between mutations and trade-offs between different properties (Bigman and Levy 2020; Li et al. 2016; Reetz et al. 2024). To preserve the high activity of *Is*PETase while improving thermostability, we discarded mutations such as W159H, S207E, and S278D, which enhanced stability but severely compromised activity (Fig. S2d, e). In contrast, such mutations (e.g., W159H and S214H) were retained in DuraPETase, likely contributing to its reduced activity under this condition (Fig. 4c). Interestingly, the thermostability-improving mutation P181V also greatly increased the soluble expression level of the enzyme (Fig. S4d, f)—a critical factor in reducing enzyme production costs. To date, studies on beneficial mutations that enhance the soluble expression of PET hydrolases remain scarce (Zhong-Johnson et al. 2024), warranting greater attention in future research. Furthermore, we also tested a suite of computational tools, including FoldX (Buss et al. 2018), Pythia (Sun et al. 2025), ThermoMPNN (Dieckhaus et al. 2024) and Catapro (Wang et al. 2025b), to predict beneficial mutations on M8. However, none of the top-ranked variants provided further improvements (Tab. S3), illustrating the ongoing limitations of purely computational design and the continued necessity for experimental screening.

Since the initiation of this work, considerable progress has been made in the mining and engineering of PET hydrolases. We therefore evaluated several newly reported, representative enzymes under our standard assay conditions (Fig. S7). These enzymes include the *Is*PETase-derived variants FAST-PETase (Tₘ = 67.1 °C) (Lu et al. 2022), and HotPETase (Tₘ = 82.5 °C) (Bell et al. 2022), TurboPETase (Tₘ = 84.0 °C) (Cui et al. 2024), CaPETase^M9^ (Tₘ = 83.2 °C) (Hong et al. 2023), Kubu-P^M12^ (Tₘ > 99.9 °C) and Mipa-P^M19^ (Tₘ = 92.4 °C) (Seo et al. 2025). Among these, only FAST-PETase exhibited higher activity than M8 (Fig. S7), suggesting that HotPETase may have sacrificed some low-temperature activity during its engineering for extreme thermostability, while other thermophilic enzymes tested were also ineffective, a trait shared with Cut190 and LCC (Fig. 1). Future studies could explore two complementary engineering strategies: introducing known stabilizing mutations into highly active scaffolds like FAST-PETase, or incorporating activity-enhancing mutations from such scaffolds into the robust M8 framework developed here.​​ These approaches may lead to more efficient depolymerases (Gao et al. 2025). Meanwhile, mining the vast natural resources—particularly marine environments—remains a promising route for discovering novel PET hydrolases. The inherently low-temperature and high-pressure conditions of the deep sea may foster the evolution of enzymes that are pre-adapted for high stability and activity under ambient conditions (Chen et al. 2024; Erickson et al. 2022).

The implementation of a fully functional SEDF process is equally contingent on the availability of a microbial chassis capable of assimilating PET-derived monomers under high-salinity conditions. Although several bacterial strains, such as *Pseudomonas* (Brandenberg et al. 2022; Werner et al. 2021) and *Rhodococcus pyridinivorans* (Narancic et al. 2021), have been reported to metabolize TPA and/or EG, ​​these studies were conducted exclusively in freshwater environments. Their adaptability and metabolic performance in high-salinity media remain largely unexplored.​ The saline lakes-derived bacterium *Halomonas bluephagenesis* (Tan et al. 2011*)*, a pioneering example of next-generation biomanufacturing, has been successfully engineered and scaled to commercial-level fermenters for polyhydroxyalkanoates (PHA) production (Park et al. 2024; Tan et al. 2021), achieving an annual capacity of tens of thousands of tons ​ (https://eng.phabuilder.com/). Thus, engineering a TPA/EG-utilizing *Halomonas* chassis represents a strategic pathway to complete the SEDF pipeline, enabling a fully operational and seawater-based bio-recycling process for PET waste.

The ​​exceptional long-term stability of *Is*PETase-M8 (retaining > 80% activity after a four-month incubation, Fig. S8)​​ under high-salinity and low-temperature conditions suggests immediate potential for applications beyond controlled bioreactors. For instance, it could be used to treat industrial wastewater contaminated with PET microplastics, such as effluent from textile processing (Deng et al. 2020). Its robustness also highlights a potential role in the in-situ bioremediation of marine PET microplastics (Huang et al. 2018; Thompson et al. 2024). However, deploying enzymes for open-environment applications like marine remediation would typically require their expression within engineered microbial chassis capable of surviving in complex ecosystems. This approach introduces substantial ecological risks and regulatory hurdles. Therefore, while M8 itself is a promising catalyst, its practical environmental deployment would necessitate extensive future studies focused on robust biocontainment strategies and comprehensive environmental impact assessments (Dvorak et al. 2017).

Finally, we note that this study was conducted at the laboratory scale (milliliter volume). A key outstanding question is whether the energy and freshwater savings of this strategy can offset its slower reaction kinetics compared to high-temperature depolymerization.​​ To address this and rigorously evaluate the industrial potential of the proposed SEDF process, future work must advance to pilot or industrial levels (e.g., cubic-meter-scale). A subsequent and essential step will be a comprehensive techno-economic analysis (TEA) comparing the operating and capital costs of this low-temperature, saline process against those of conventional high-temperature, freshwater-based depolymerization. This analysis should specifically account for large-scale enzyme production costs, quantify the energy savings from eliminating heating and freshwater purification steps, and compare reactor capital costs under the two distinct operating regimes. Only through such scaled experimentation and detailed economic modeling can the true economic competitiveness and practical viability of this approach be definitively established.

In conclusion, we have engineered a robust PET hydrolase variant *Is*PETase-M8, which exhibits simultaneous and substantial enhancements in thermostability (ΔT_m_ = + 27.3 °C), activity (1.14-fold increase), and soluble expression yield (14.3-fold increase). Its overall depolymerization efficiency surpassed that of the thermostable benchmarks DuraPETase and LCC-ICCG by ​​32.2- and 10.4-fold,​​ respectively, under identical conditions. Most notably, M8 achieved continuous depolymerization of PET at ​​15% (w/v) solid loading​​ in natural seawater (37 °C), yielding monomers at a rate of 15.4 mM/day—a concentration sufficient to support downstream microbial assimilation. This work establishes an efficient enzymatic platform and provides a technological foundation for implementing SEDF processes entirely in saline environments. By enabling energy-efficient, freshwater-free bioconversion, our study aligns with the NGIB principles and presents a practical strategy for sustainable plastic bio-recycling.

## Supplementary Information

Supplementary Material 1.

## Data Availability

The atomic coordinates and structure factors of the reported structure have been deposited in the Protein Data Bank under accession code 7VWN for *Is*PETase-M8. All data supporting the findings of this study are available within the paper and its Supplementary Information.

## Abbreviations

PET: Poly(ethylene terephthalate)
TPA: Terephthalic acid
EG: Ethylene glycol
MHET: Mono-(2-hydroxyethyl) terephthalate
BHET: Bis(2-hydroxyethyl) terephthalate
2-HE(MHET)_5_: 2-Hydroxyethyl-(monohydroxyethyl terephthalate)_5_
T_g_: Glass transition temperature
SEDF: Simultaneous enzymatic depolymerization and fermentation
SSF: Simultaneous saccharification and fermentation
NGIB: Next-generation industrial biotechnology
*E. coli*: *Escherichia coli*
X-C_2_: 5-Bromo-4-chloro-3-indolyl acetate
X-C_8_: 5-Bromo-4-chloro-3-indolyl octanoate
pNP-acetate: *para*-Nitrophenyl-acetate
SM: Saturation mutagenesis
WT: Wild-type
T_m_: Melting temperature
HPLC: High performance liquid chromatography
SDS-PAGE: Sodium dodecyl sulfate polyacrylamide gel electrophoresis
WB: Western blot
MD: Molecular dynamics
RMSD: Root mean square deviation
RMSF: Root mean square fluctuations
