# Supplementary material for: Engineering a robust IsPETase for energy-efficient PET depolymerization in natural seawater at ambient temperatures
Source: Adv Biotechnol (Singap). 2026 Apr 15;4(2):14. doi: 10.1007/s44307-026-00104-z (PMC13083688; doi:10.1007/s44307-026-00104-z)
Supplement: Supplementary file 1 — Supplementary Material 1. [file 44307_2026_104_MOESM1_ESM.docx]

**Supplementary information**

**Engineering a robust IsPETase variant for energy-efficient PET depolymerization in natural seawater at ambient temperatures**

Xin Huang, Qian Jia, Guang Li, Xiangpeng Yang, Shujing Xu, Jianzhong Liu, Wenjun Li, Yuhuan Liu, Wei Xie*, Lichuang Cao*

*State Key Laboratory of Biocontrol, Innovation Center for Evolutionary Synthetic Biology,* *Guangzhou Innovation Center of Biotechnology and Biomanufacturing, School of Life Sciences, Sun Yat-Sen University, Guangzhou, 510275, China*

*Corresponding author:

Wei Xie (E-mail: caolch5@mail.sysu.edu.cn)

Lichuang Cao (E-mail: caolch5@mail.sysu.edu.cn)

Tel: 86-20-84113712

Fax: 86-20-84036215


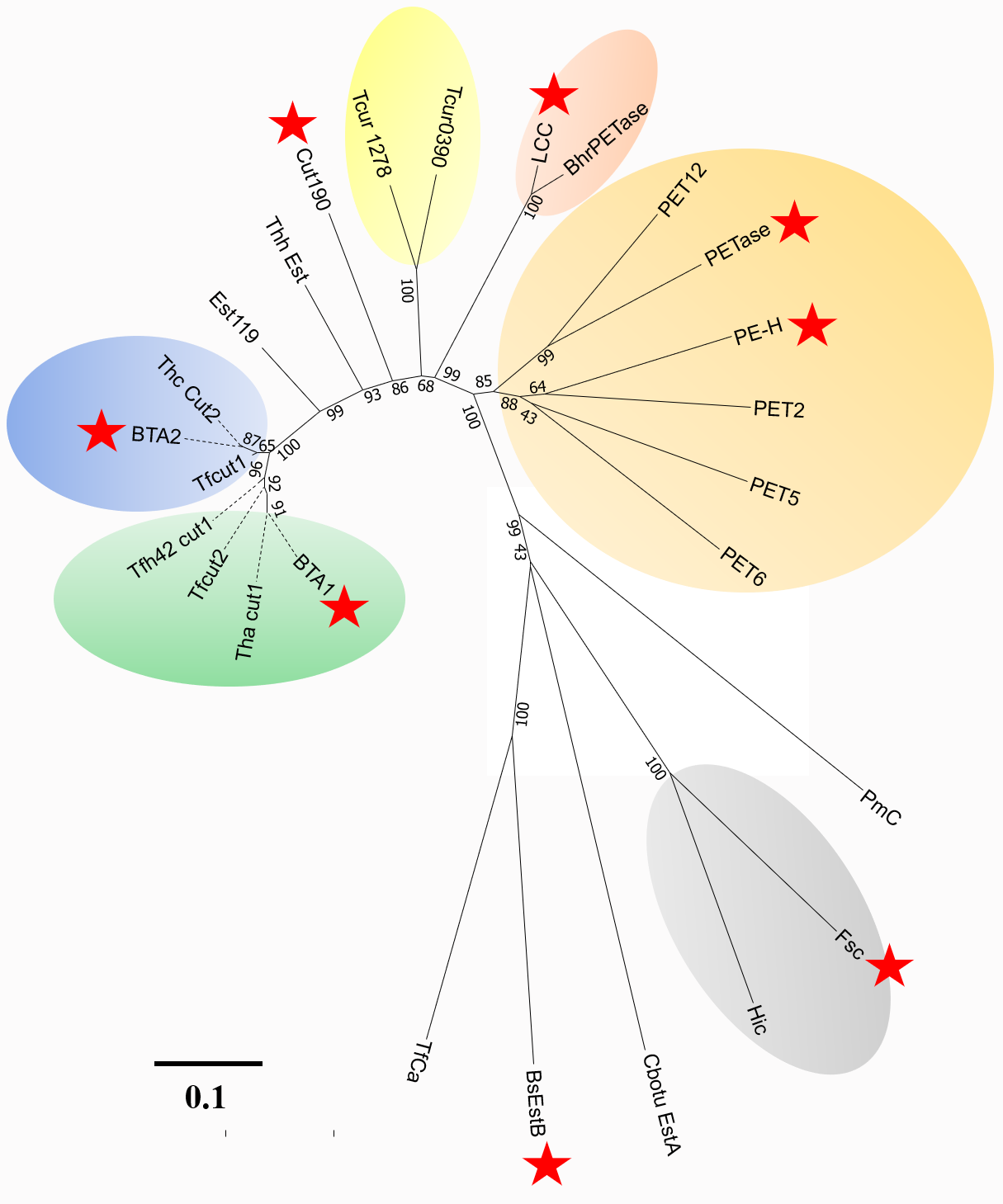


**Fig. S1 Phylogenetic tree of the 26 reported PET-hydrolyzing enzymes.** The amino acid sequences were obtained from UniProt or Genbank database. The accession numbers are: BhrPETase, GBD22443(Genbank); BsEstB, D7R6G8 (UniProt); BTA1, Q6A0I4 (UniProt); BTA2, Q6A0I3 (UniProt); Cut190, W0TJ64 (UniProt); Cbotu_EstA; AKZ20828.1(genebank); Est119, F7IX06 (UniProt); FsC, P00590 (UniProt); HiC, A0A075B5G4 (UniProt); *Is*PETase, A0A0K8P6T7 (UniProt); Tcur_1278, D1A9G5 (UniProt); TfCa, C5JAB7 (UniProt); TfCut2, E5BBQ3 (UniProt); Tfh42_Cut1, E9LVI0 (UniProt); Tha_Cut1, E9LVH7 (UniProt); TfCut1, E5BBQ2 (UniProt); Thc_Cut2, E9LVH9 (UniProt); Thh_Est, H6WX58 (UniProt); LCC, G9BY57 (UniProt); PE-H, A0A1H6AD45 (UniProt); PET2, C3RYL0 (UniProt); PET5, R4YKL9 (UniProt); PET6, UPI0003945E1F (UniProt); PET12, A0A0G3BI90 (UniProt); Tcur_0390, CDN67546.1 (Genebank) and PmC, A4Y035 (UniProt). Multiple sequence alignment was performed by ClustalW. The tree was generated by MEGAX (https://www.megasoftware.net/) using the neighbor-joining algorithm. Bootstrap values were shown at each node as percentage of 1000 replicates. The closely related enzymes were grouped by different colors. Scale bar, 0.1 amino acid substitutions per single site. Several enzymes were connected to the actual branch by dotted lines for viewing convenience. The enzymes characterized in this study were marked by red stars.


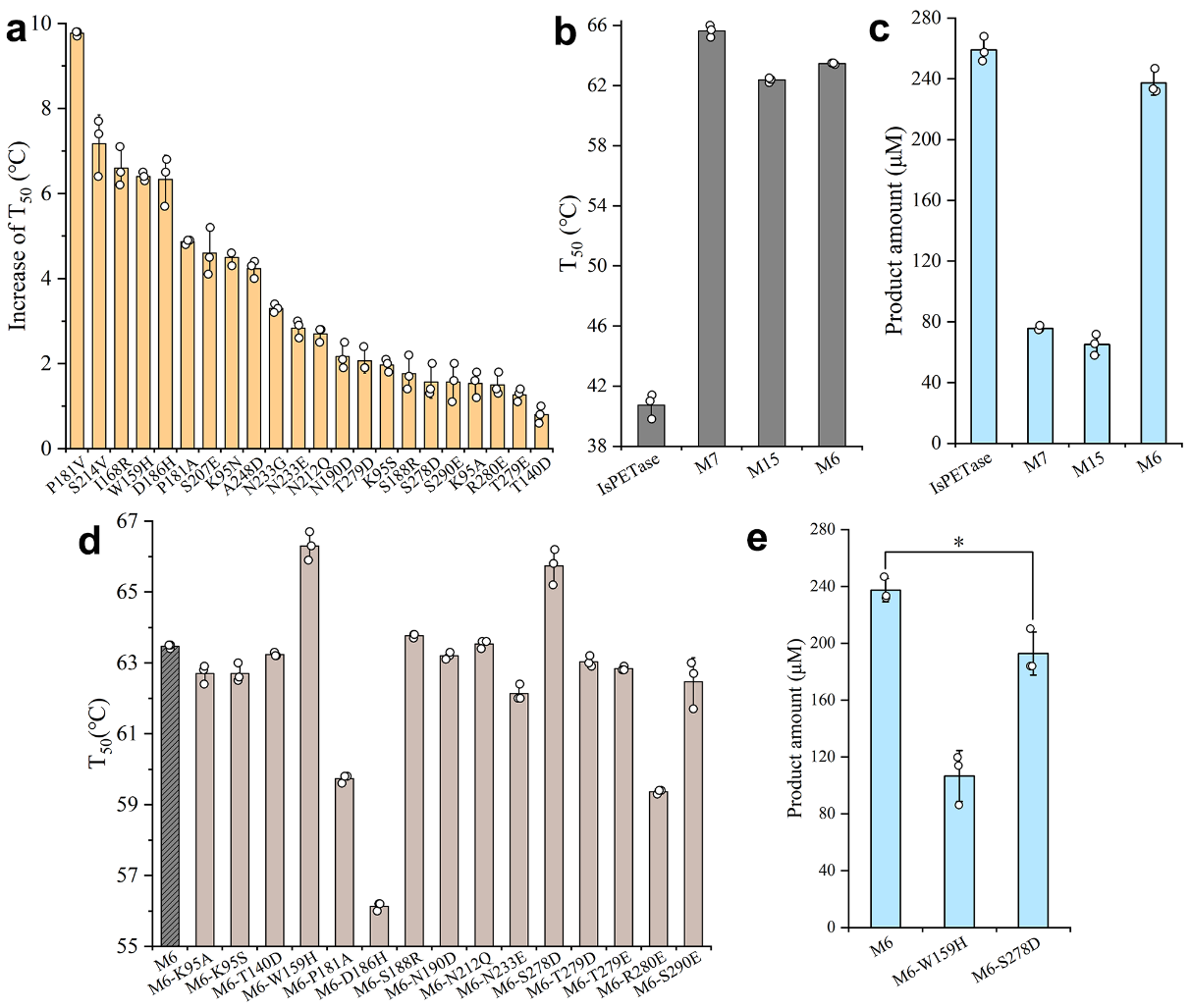


**Fig. S2 Thermostability and hydrolysis activity of the *Is*PETase mutants.** (a) T_50_ increases of the 22 single point mutants compared to wild-type *Is*PETase. (b) T_50_ values of multi-mutants M7, M15 and M6. (c) Hydrolysis activities of M7, M15 and M6. (d) T_50_ values of M6-based mutants. (e) Hydrolysis activities of mutants M6-W159H and M6-S278D. *: The *P* value is 0.0055 (one-sided t-test). The bars and cycles are the average and individual numbers of triplicate measurements, respectively. The error bars represent standard deviation.


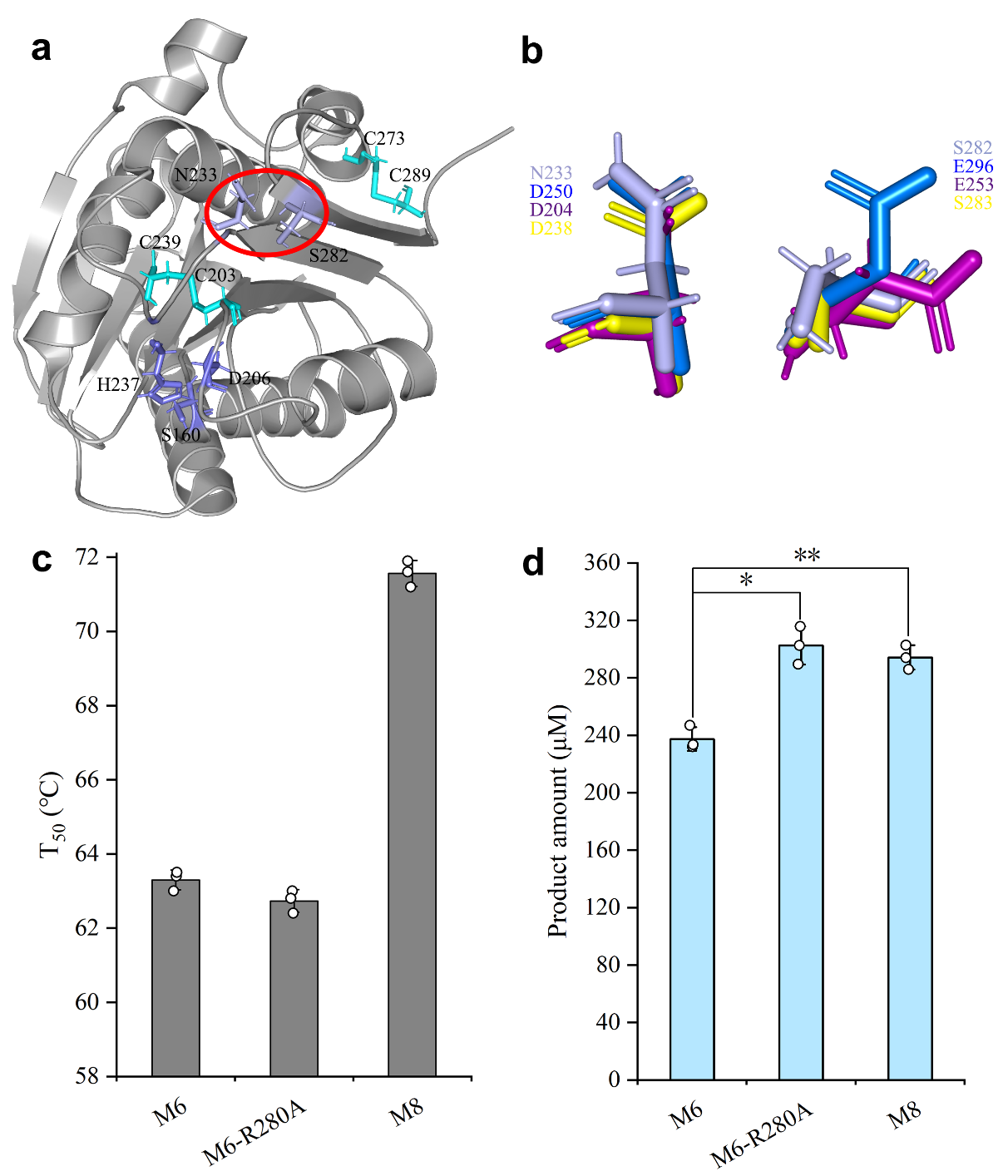


**Fig. S3 Improvement on thermostability and hydrolysis activity of mutant M6 by rational design.** (a) Location of residues for the introduced disulfide bond (N233 and S282, light blue sticks, marked by a red cycle) in *Is*PETase (PDB 6EQE). The catalytic triad (S160, D206 and H237, slate sticks) and two original disulfide bonds (C203-C239 and C273-C289, cyan sticks) of *Is*PETase were also shown. (b) The disulfide bond was introduced based on the results of identified PET hydrolases, including Cut190 from *Saccharomonospora viridis* (PDB 5ZNO, blue), TfCut2 from *Thermobifida fusca* KW3 (PDB 4CG1, purple) and LCC from a metagenomic library (PDB 6THS, yellow). (c) Effects of R280A and disulfide bond G233C-S282C on the T_50_ of mutant M6. (d) Effects of R280A and disulfide bond G233C-S282C on the hydrolysis activity of mutant M6. M8: M6+R280A+G233C+S282C. Please note that M6 already contains the mutation N233G. Statistical analyses were performed using a one-sided t-test. The *P* values were 0.0009 (*) and 0.0006 (**). The bars and cycles are the average and individual numbers of triplicate measurements, respectively. The error bars represent standard deviation.


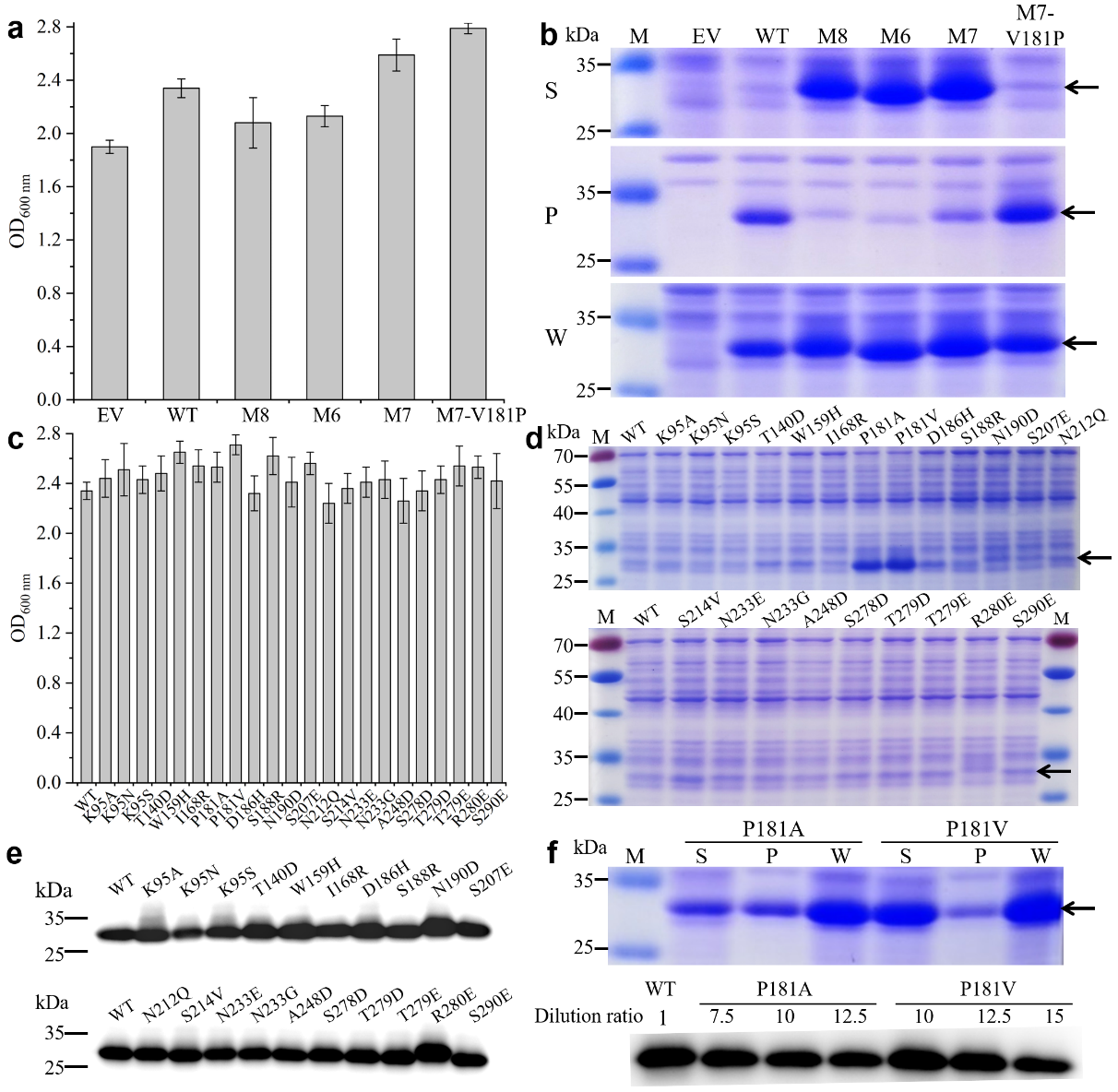


**Fig. S4 Soluble expression of *Is*PETase and its mutants.** (a) The optical density at 600 nm (OD_600 nm_) of *Is*PETase and mutants. The bars are the average numbers of three biological replicates, and the error bars represent standard deviation. (b) Sodium dodecyl sulfate polyacrylamide gel electrophoresis (SDS-PAGE) analyses of the whole cell (W), supernatants (S) and pellets (P) of *Is*PETase and mutants. (c) OD_600 nm_ of the 22 single point mutants. The bars are the average numbers of three biological replicates, and the error bars represent standard deviation. (d) SDS-PAGE analyses of the supernatants of 22 single point mutants. (e) Western blot analyses of the 20 single point mutants. (f) SDS-PAGE and western blot analyses of P181A and P181V. Gray analyses of the bands in western blots (three biological replicates) showed that the soluble expression yields of P181A and P181V were 7.5- and 12.3-folds that of *Is*PETase, respectively. Abbreviations: kDa, kilodaltons; EV, empty vector; WT, wild-type *Is*PETase, M: marker. The specific bands of the target protein were marked by arrows. All the proteins were induced by 0.6 mM isopropyl β-D-thiogalactopyranoside (IPTG) at 16 ℃ for 24 h. See methods for details about the conditions of cultivation, induction, sample preparation and experimental protocol. Uncropped gels and blots are provided in Fig. S8.


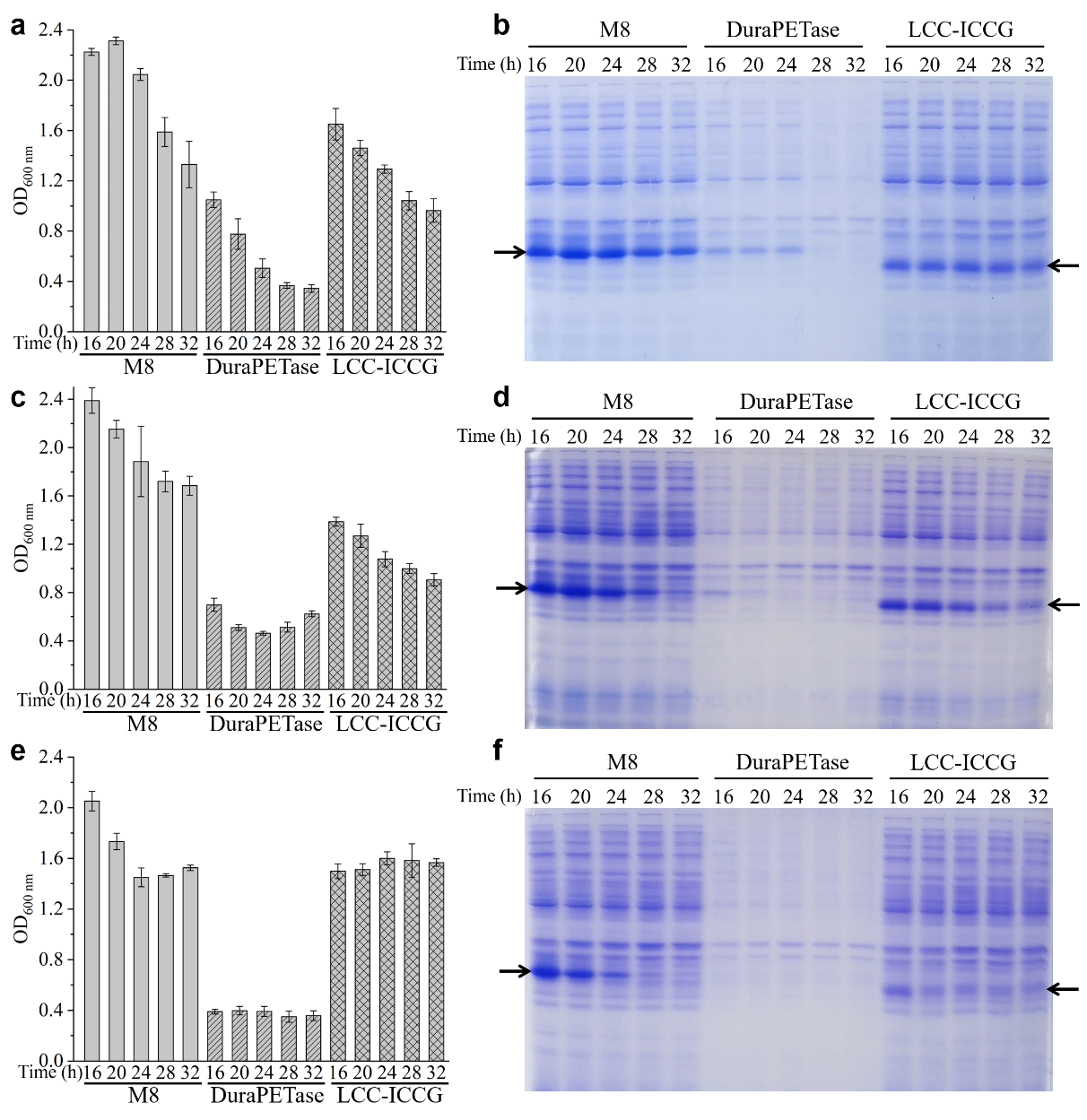


**Fig. S5 The soluble expression of M8, DuraPETase and LCC-ICCG.** The proteins were induced by 0.6 mM isopropyl β-D-thiogalactopyranoside (IPTG) at 16 (a, b), 20 (c, d) and 25 ℃ (e, f) for different time. The optical density at 600 nm (OD_600 nm_) was determined. Supernatants of the samples were analyzed by sodium dodecyl sulfate polyacrylamide gel electrophoresis (SDS-PAGE). Obviously, the soluble expression yield of M8 is much higher than that of DuraPETase and LCC-ICCG. The expression level of DuraPETase was very low under the conditions of >24 h at 16 ℃, >16 h at 20 ℃ and 25 ℃, so we selected the samples of 24 h at 16 ℃ to compare their soluble expression yield (Fig. 4). In addition, the overexpression of DuraPETase and LCC-ICCG inhibited the growth of host cells, especially DuraPETase, whose OD_600 nm_ after induction was even lower than that before the induction (~0.8). The bars are the average numbers of three biological replicates, and the error bars represent standard deviation. The target protein bands were marked by black arrows. See methods for experimental details.


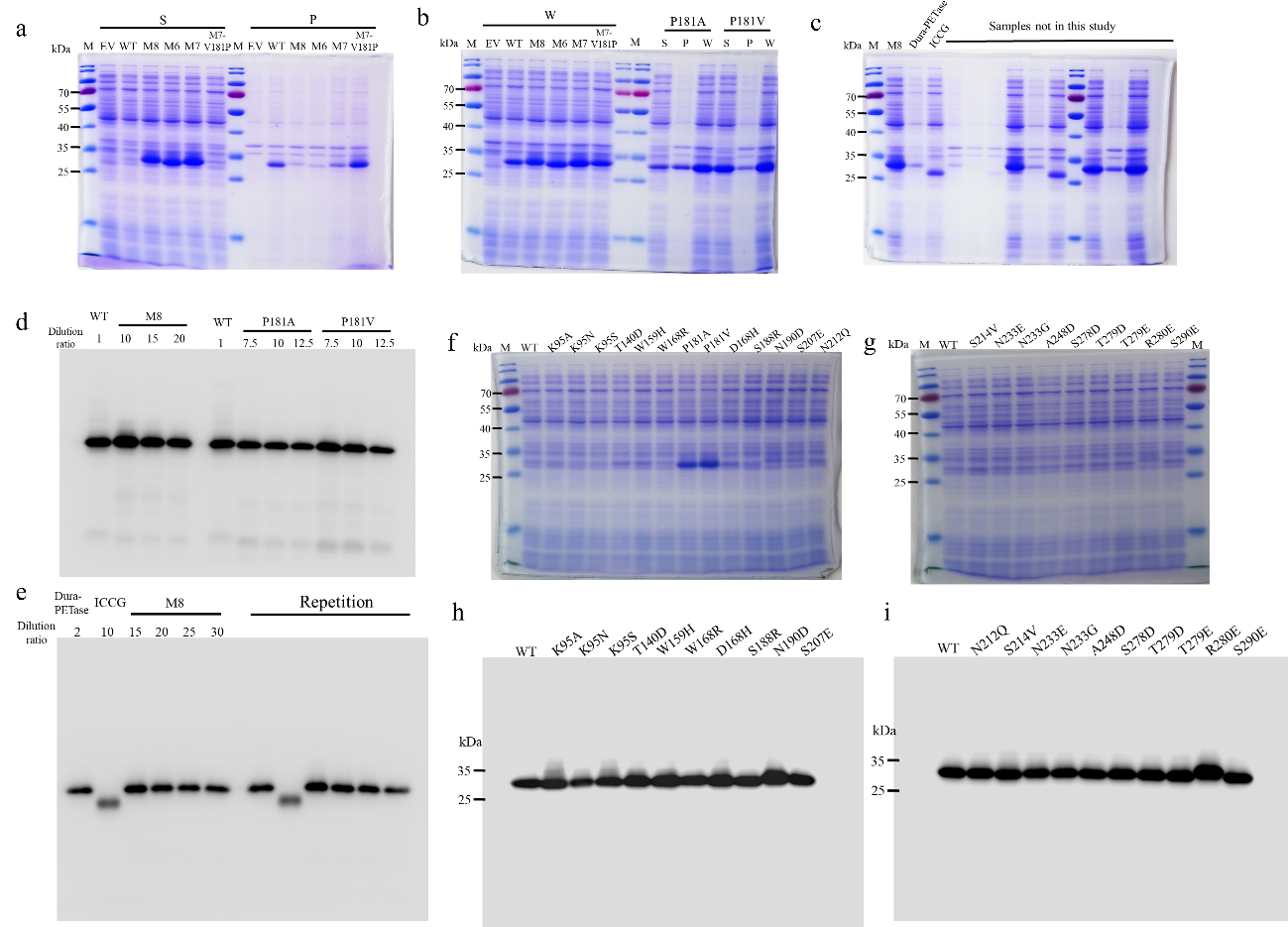


**Fig. S6 Uncropped gels and blots images.** (a, b) SDS-PAGE analyses of *Is*PETase and its mutants: Supernatants (S), pellets (P) and Whole cell (W). (c) SDS-PAGE analysis of the soluble expression of M8, DuraPETase and LCC-ICCG. (d) Western blot analyses of M8 and key mutants P181A and P181V. (e) Western blot analyses of M8, DuraPETase and LCC-ICCG. (f-i) Expression characterization of 22 single-point mutants: SDS-PAGE analyses of soluble expression (f, g) and western blot analyses of corresponding single-point mutants (h, i).

**
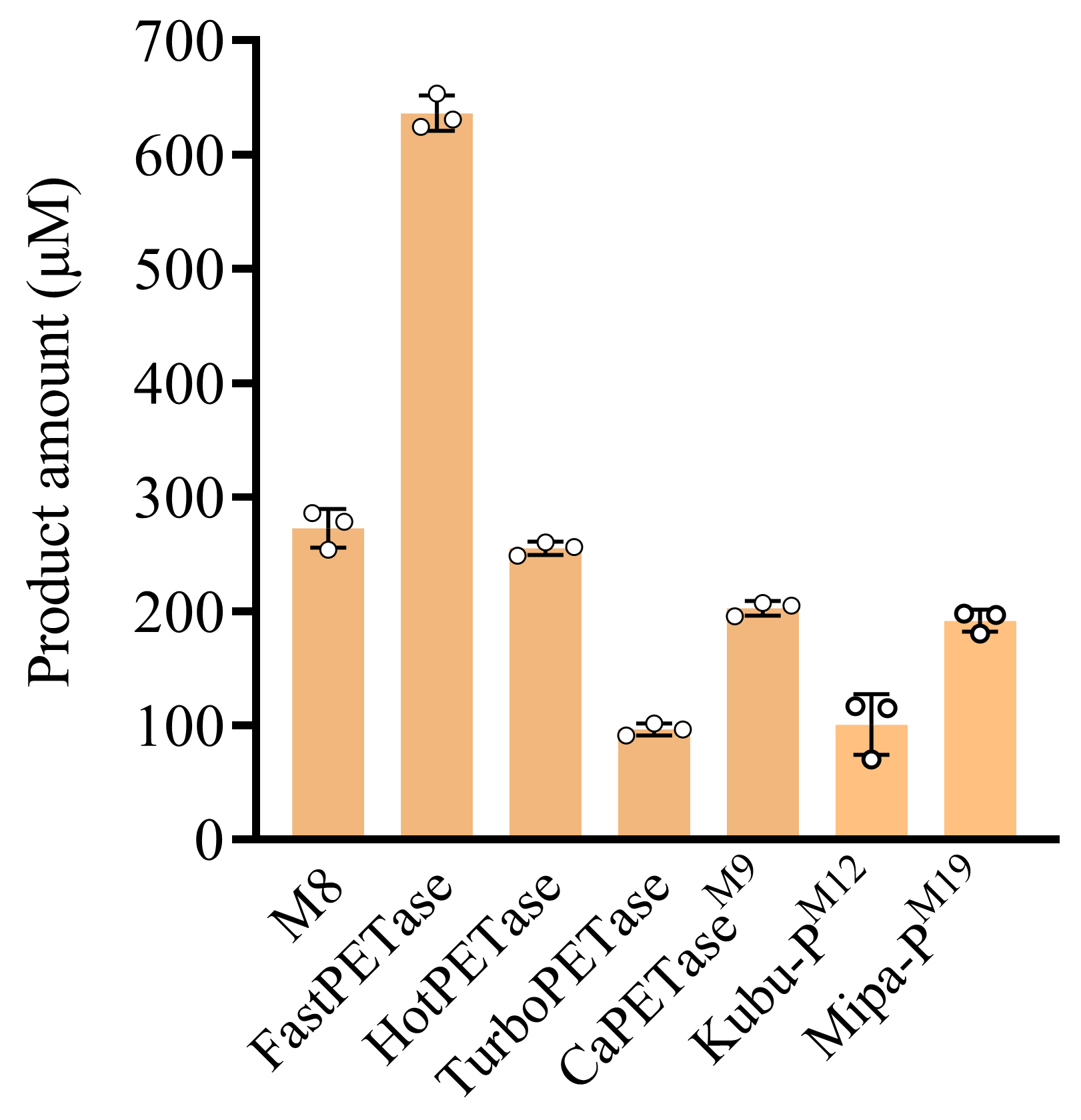
**

**Fig. S7 Activity comparison of several PET hydrolases in artificial seawater.** The reactions were performed at 30 ℃ for 18 h with 500 nM purified proteins. The bars and cycles are the average and individual numbers of triplicate measurements, respectively. The error bars represent standard deviation.

**
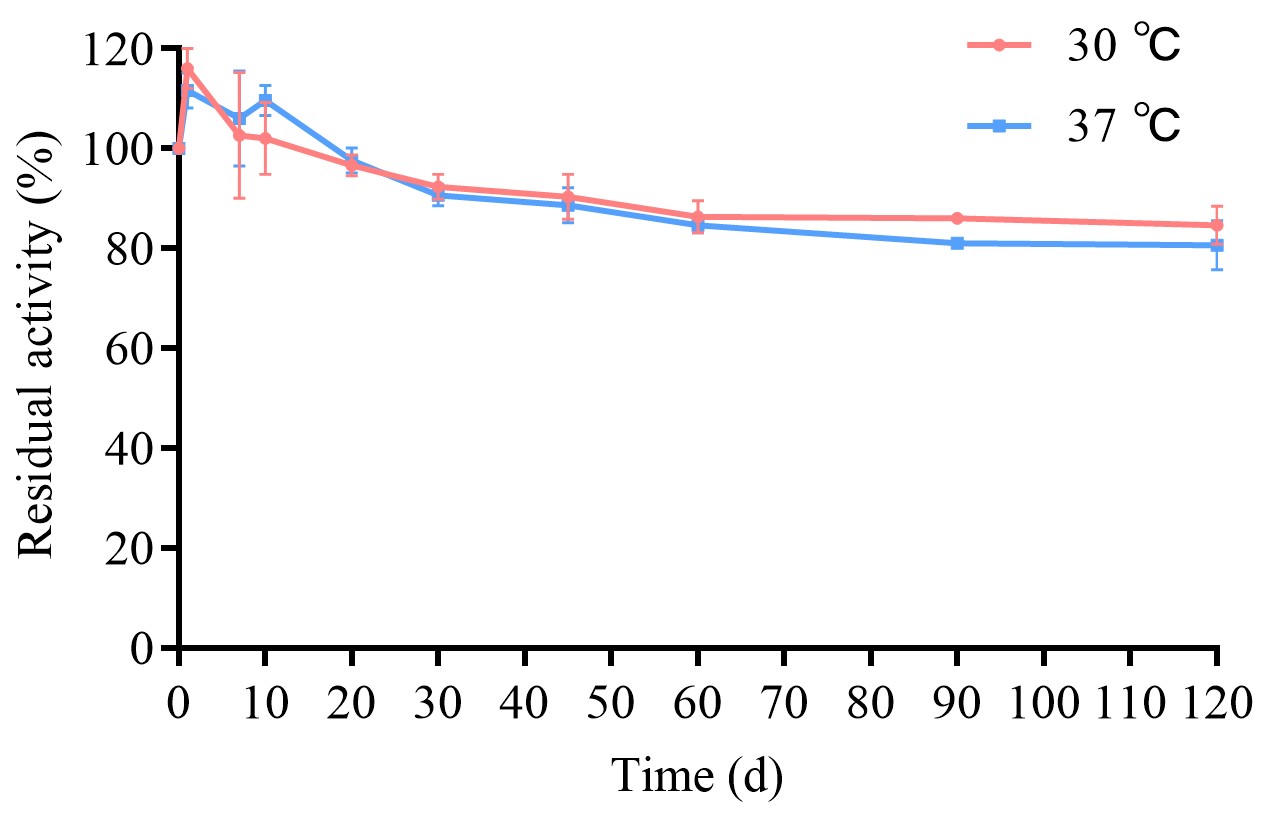
**

**Fig. S8 The residual activity of mutant M8 during the incubation in seawater at 30℃ and 37℃.** Purified M8 was diluted to 500 nM in seawater with 50 mM Tris-HCl buffers and then was incubated at 30℃ and 37℃. At different time intervals, the residual activity towards *para*-nitrophenyl (pNP)-acetate was measured. Data points are the average of triplicate measurements, where error bars represent standard deviation.

**Tab. S1 Primers used in this study**

| Primers used to construct mutants of LCC | | |  |
| --- | --- | --- | --- |
| Templates | Mutations | Primers | |
| LCC | Y127G | FP: **GGC**CCGGACAGCCGCGCCTCTCAGCTGAG | |
|  |  | RP: ATCGAAACGTGAATTCGTGTTAATCACC | |
| LCC-Y127G | D238C/F243I | FP: **TGC**AACGCATCGCAC**ATT**GCTCCGAATAGC | |
|  |  | RP: CAGTTCGACGTAAACTTTCGGCGTGGTGC | |
| LCC-Y127G/  D238C/F243I | S283C | FP: **TGC**GACTTCCGCACCAATAATCGCCAC | |
|  |  | RP: CAGAGCCGGGTCGTTCACATTACACAGG | |
| Primers used to construct saturation mutagenesis libraries of *Is*PETase | | |  |
| Templates | Mutations | Primers | |
| *Is*PETase | N30-NNK | FP: **NNK**CCGTACGCGCGTGGTCCAAATCCAA | |
|  |  | RP: GGATCCCATATGTATATCTCCTTCTTAAAGT | |
|  | P31-NNK | FP: **NNK**TACGCGCGTGGTCCAAATCCAACCGC | |
|  |  | RP: ATTGGATCCCATATGTATATCTCCTTCTTAA | |
|  | P36-NNK | FP: **NNK**AATCCAACCGCCGCGAGTCTGGAAGC | |
|  |  | RP: ACCACGCGCGTACGGATTGGATCCCATATG | |
|  | N37-NNK | FP: **NNK**CCAACCGCCGCGAGTCTGGAAGCGAG | |
|  |  | RP: TGGACCACGCGCGTACGGATTGGATCCCAT | |
|  | P38-NNK | FP: **NNK**ACCGCCGCGAGTCTGGAAGCGAGTGC | |
|  |  | RP: ATTTGGACCACGCGCGTACGGATTGGATCC | |
|  | S46-NNK | FP: **NNK**GCCGGTCCATTCACCGTTCGCAGCT | |
|  |  | RP: CGCTTCCAGACTCGCGGCGGTTGGATTT | |
|  | A47-NNK | FP: **NNK**GGTCCATTCACCGTTCGCAGCTTTA | |
|  |  | RP: ACTCGCTTCCAGACTCGCGGCGGTTG | |
|  | V57-NNK | FP: **NNK**AGCCGCCCAAGCGGTTATGGCGCC | |
|  |  | RP: GGTAAAGCTGCGAACGGTGAATGGACC | |
|  | S58-NNK | FP: **NNK**CGCCCAAGCGGTTATGGCGCCGGCA | |
|  |  | RP: AACGGTAAAGCTGCGAACGGTGAATGG | |
|  | R59-NNK | FP: **NNK**CCAAGCGGTTATGGCGCCGGCACCG | |
|  |  | RP: GCTAACGGTAAAGCTGCGAACGGTGAA | |
|  | P60-NNK | FP: **NNK**AGCGGTTATGGCGCCGGCACCGTGT | |
|  |  | RP: GCGGCTAACGGTAAAGCTGCGAACGGT | |
|  | T72-NNK | FP: **NNK**AATGCGGGTGGCACCGTGGGTGC | |
|  |  | RP: CGGGTAGTACACGGTGCCGGCGCCA | |
|  | N73-NNK | FP: **NNK**GCGGGTGGCACCGTGGGTGCCAT | |
|  |  | RP: GGTCGGGTAGTACACGGTGCCGGCG | |
|  | A74-NNK | FP: **NNK**GGTGGCACCGTGGGTGCCATTGCGA | |
|  |  | RP: ATTGGTCGGGTAGTACACGGTGCCG | |
|  | G75-NNK | FP: **NNK**GGCACCGTGGGTGCCATTGCGATTG | |
|  |  | RP: CGCATTGGTCGGGTAGTACACGGTG | |
|  | K95-NNK | FP: **NNK**TGGTGGGGTCCACGTCTGGCGAGTCA | |
|  |  | RP: GATGCTGCTCTGACGGGCGGTATAAC | |
|  | L117-NNK | FP: **NNK**GACCAGCCGAGCAGTCGCAGTAGCC | |
|  |  | RP: CGTGCTGTTGGTATCGATGGTGATC | |
|  | Q119-NNK | FP: **NNK**CCGAGCAGTCGCAGTAGCCAACAG | |
|  |  | RP: GTCCAGCGTGCTGTTGGTATCGATG | |
|  | T140-NNK | FP: **NNK**AGCAGCAGCCCAATCTACGGCAAAG | |
|  |  | RP: GCCATTCAGACTCGCAACTTGGCGC | |
|  | S141-NNK | FP: **NNK**AGCAGCCCAATCTACGGCAAAGTGG | |
|  |  | RP: GGTGCCATTCAGACTCGCAACTTGG | |
|  | S142-NNK | FP: **NNK**AGCCCAATCTACGGCAAAGTGGATA | |
|  |  | RP: GCTGGTGCCATTCAGACTCGCAACT | |
|  | S143-NNK | FP: **NNK**CCAATCTACGGCAAAGTGGATACCG | |
|  |  | RP: GCTGCTGGTGCCATTCAGACTCGCA | |
|  | Y146-NNK | FP: **NNK**GGCAAAGTGGATACCGCGCGCATGG | |
|  |  | RP: GATTGGGCTGCTGCTGGTGCCATTC | |
|  | K148-NNK | FP: **NNK**GTGGATACCGCGCGCATGGGCGTG | |
|  |  | RP: GCCGTAGATTGGGCTGCTGCTGGTG | |
|  | W159-NNK | FP: **NNK**AGTATGGGTGGTGGCGGCAGTCT | |
|  |  | RP: GCCCATCACGCCCATGCGCGCGGTA | |
|  | I168-NNK | FP: **NNK**AGCGCCGCCAATAACCCAAGTCTG | |
|  |  | RP: CAGACTGCCGCCACCACCCATACTC | |
|  | K177-NNK | FP: **NNK**GCCGCCGCGCCACAAGCGCCGTGGG | |
|  |  | RP: CAGACTTGGGTTATTGGCGGCGCTA | |
|  | P181-NNK | FP: **NNK**CAAGCGCCGTGGGATAGTAGCACC | |
|  |  | RP: CGCGGCGGCTTTCAGACTTGGGTTA | |
|  | W185-NNK | FP: **NNK**GATAGTAGCACCAACTTCAGCAGT | |
|  |  | RP: CGGCGCTTGTGGCGCGGCGGCTTTC | |
|  | D186-NNK | FP: **NNK**AGTAGCACCAACTTCAGCAGTGTG | |
|  |  | RP: CCACGGCGCTTGTGGCGCGGCGGCT | |
|  | S187-NNK | FP: **NNK**AGCACCAACTTCAGCAGTGTGACGG | |
|  |  | RP: ATCCCACGGCGCTTGTGGCGCGGCG | |
|  | S188-NNK | FP: **NNK**ACCAACTTCAGCAGTGTGACGGTTC | |
|  |  | RP: ACTATCCCACGGCGCTTGTGGCGCG | |
|  | T189-NNK | FP: **NNK**AACTTCAGCAGTGTGACGGTTCCG | |
|  |  | RP: GCTACTATCCCACGGCGCTTGTGGC | |
|  | N190-NNK | FP: **NNK**TTCAGCAGTGTGACGGTTCCGACG | |
|  |  | RP: GGTGCTACTATCCCACGGCGCTTGT | |
|  | S193-NNK | FP: **NNK**GTGACGGTTCCGACGCTGATCTTTG | |
|  |  | RP: GCTGAAGTTGGTGCTACTATCCCAC | |
|  | N205-NNK | FP: **NNK**GACAGCATCGCGCCAGTGAATAGCA | |
|  |  | RP: TTCGCACGCAAAGATCAGCGTCGGA | |
|  | S207-NNK | FP: **NNK**ATCGCGCCAGTGAATAGCAGCGCCC | |
|  |  | RP: GTCGTTTTCGCACGCAAAGATCAGC | |
|  | N212-NNK | FP: **NNK**AGCAGCGCCCTCCCGATCTACGAT | |
|  |  | RP: CACTGGCGCGATGCTGTCGTTTTCG | |
|  | S213-NNK | FP: **NNK**AGCGCCCTCCCGATCTACGATAG | |
|  |  | RP: ATTCACTGGCGCGATGCTGTCGTTT | |
|  | S214-NNK | FP: **NNK**GCCCTCCCGATCTACGATAGCATG | |
|  |  | RP: GCTATTCACTGGCGCGATGCTGTC | |
|  | S223-NNK | FP: **NNK**CGCAATGCGAAGCAGTTTCTGGA | |
|  |  | RP: CATGCTATCGTAGATCGGGAGGGCG | |
|  | R224-NNK | FP: **NNK**AATGCGAAGCAGTTTCTGGAGAT | |
|  |  | RP: GCTCATGCTATCGTAGATCGGGAGG | |
|  | N233-NNK | FP: **NNK**GGCGGTAGCCATAGCTGCGCCAAC | |
|  |  | RP: GATCTCCAGAAACTGCTTCGCATTG | |
|  | S245-NNK | FP: **NNK**AATCAAGCGCTGATCGGCAAGAA | |
|  |  | RP: GTTACCGCTGTTGGCGCAGCTATGG | |
|  | A248-NNK | FP: **NNK**CTGATCGGCAAGAAAGGCGTTGCG | |
|  |  | RP: TTGATTGCTGTTACCGCTGTTGGCG | |
|  | N275-NNK | FP: **NNK**CCGAATAGTACGCGCGTGAGCGAC | |
|  |  | RP: CTCGCACGCAAACGTGCTGTAGCGG | |
|  | P276-NNK | FP: **NNK**AATAGTACGCGCGTGAGCGACTT | |
|  |  | RP: ATTCTCGCACGCAAACGTGCTGTAG | |
|  | N277-NNK | FP: **NNK**AGTACGCGCGTGAGCGACTTT | |
|  |  | RP: CGGATTCTCGCACGCAAACGTGCTG | |
|  | S278-NNK | FP: **NNK**ACGCGCGTGAGCGACTTTCGTACC | |
|  |  | RP: ATTCGGATTCTCGCACGCAAACGTG | |
|  | T279-NNK | FP: **NNK**CGCGTGAGCGACTTTCGTACCGCG | |
|  |  | RP: ACTATTCGGATTCTCGCACGCAAAC | |
|  | R280-NNK | FP: **NNK**GTGAGCGACTTTCGTACCGCGAATT | |
|  |  | RP: CGTACTATTCGGATTCTCGCACGCAA | |
|  | S282-NNK | FP: **NNK**GACTTTCGTACCGCGAATTGCAGC | |
|  |  | RP: CACGCGCGTACTATTCGGATTCTCG | |
|  | D283-NNK | FP: **NNK**TTTCGTACCGCGAATTGCAGC | |
|  |  | RP: GCTCACGCGCGTACTATTCGGATTC | |
|  | C289-NNK | FP: **NNK**AGCCTCGAGCACCACCACCACCACCA | |
|  |  | RP: ATTCGCGGTACGAAAGTCGCTCACG | |
|  | S290-NNK | FP: **NNK**CTCGAGCACCACCACCACCACCACTG | |
|  |  | RP: GCAATTCGCGGTACGAAAGTCGCTC | |
| Primers used to construct single point mutants of *Is*PETase | | |  |
| Templates | Mutations | Primers | |
| M7 | E207S | FP: **AGC**ATCGCGCCAGTGAATAGCGTGGC | |
|  |  | RP: GTCGTTTTCGCACGCAAAGATCAGC | |
| M6 | N95A | FP: **GCG**TGGTGGGGTCCACGTCTGGCGAG | |
|  |  | RP: GATGCTGCTCTGACGGGCGGTATAACC | |
|  | N95S | FP: **TCT**TGGTGGGGTCCACGTCTGGCGAG | |
|  |  | RP: GATGCTGCTCTGACGGGCGGTATAACC | |
|  | N140D | FP: **GAT**AGCAGCAGCCCAATCTACGGCAAA | |
|  |  | RP: GCCATTCAGACTCGCAACTTGGCGC | |
|  | W159H | FP: **CAT**AGTATGGGTGGTGGCGGCAGTCT | |
|  |  | RP: GCCCATCACGCCCATGCGCGCGGTAT | |
|  | V181A | FP: **GCT**CAAGCGCCGTGGGATAGTAGCA | |
|  |  | RP: CGCGGCGGCTTTCAGACTTGGGTTA | |
|  | D186H | FP: **CAT**AGTAGCACCAACTTCAGCAGTGT | |
|  |  | RP: CCACGGCGCTTGCACCGCGGCGGCT | |
|  | S188R | FP: **CGT**ACCAACTTCAGCAGTGTGACGGTT | |
|  |  | RP: ACTATCCCACGGCGCTTGCACCGCGG | |
|  | N190D | FP: **GAT**TTCAGCAGTGTGACGGTTCCGA | |
|  |  | RP: GGTGCTACTATCCCACGGCGCTTG | |
|  | N212Q | FP: **CAG**AGCGTGGCCCTCCCGATCTACGAT | |
|  |  | RP: CACTGGCGCGATGCTGTCGTTTTCGC | |
|  | G233E | FP: **GAG**GGCGGTAGCCATAGCTGCGCCA | |
|  |  | RP: GATCTCCAGAAACTGCTTCGCATTG | |
|  | S278D | FP: **GAT**ACGCGCGTGAGCGACTTTCGTACC | |
|  |  | RP: ATTCGGATTCTCGCACGCAAACGT | |
|  | T279D | FP: **GAT**CGCGTGAGCGACTTTCGTACCGCGA | |
|  |  | RP: ACTATTCGGATTCTCGCACGCAAACG | |
|  | T279E | FP: **GAG**CGCGTGAGCGACTTTCGTACCGCGA | |
|  |  | RP: ACTATTCGGATTCTCGCACGCAAACG | |
|  | R280E | FP: **GAG**GTGAGCGACTTTCGTACCGCGAA | |
|  |  | RP: CGTACTATTCGGATTCTCGCACGC | |
|  | S290E | FP: **GAG**CTCGAGCACCACCACCACCACCACTG | |
|  |  | RP: GCAATTCGCGGTACGAAAGTCGCTCA | |
|  | R280A | FP: **GCT**GTGAGCGACTTTCGTACCGCGAA | |
|  |  | RP: CGTACTATTCGGATTCTCGCACGC | |
| M6-R280A | G233C | FP: **TGC**GGCGGTAGCCATAGCTGCGCCA | |
|  |  | RP: GATCTCCAGAAACTGCTTCGCATTG | |
| M6-R280A/  G233C | S282C | FP: GCTGTG**TGC**GACTTTCGTACCGCGAA | |
|  |  | RP: CGTACTATTCGGATTCTCGCACGC | |

**Tab. S2 Data collection and refinement statistics**

|  | *Is*PETase-M8 |
| --- | --- |
| **Data collection** |  |
| Space group | *C*121 |
| Cell dimensions |  |
| *a*, *b*, *c* (Å) | 115.59, 50.54, 41.37 |
| α, β, γ (°) | 90.00, 92.46, 90.00 |
| Resolution (Å) | 50.00-1.45 (1.50-1.45)* |
| *R*_merge_ | 0.063 (0.38) |
| *I* / σ*I* | 23.3 (2.8) |
| Completeness (%) | 98.2 (91.0) |
| Redundancy | 6.7 (6.0) |
|  |  |
| **Refinement** |  |
| Resolution (Å) | 32.96-1.45 (1.49-1.45) |
| No. reflections | 41548 |
| *R*_work_ / *R*_free_ | 0.155/0.179 |
| No. atoms |  |
| Protein | 1936 |
| Ligand/ion | 3 |
| Water | 282 |
| *B*-factors |  |
| Protein | 21.4 |
| Ligand/ion | 32.2 |
| Water | 40.6 |
| R.m.s. deviations |  |
| Bond lengths (Å) | 0.014 |
| Bond angles (°) | 1.39 |

*Values in parentheses are for highest-resolution shell. The data were collected from a single crystal.

**Tab. S3 Top-ranked mutations predicted by computational tools for the M8 variant​**

| Pythia | | ThermoMPNN | | Foldx | | Catapro | |
| --- | --- | --- | --- | --- | --- | --- | --- |
| Mutation | ΔΔG | Mutation | ΔΔG | Mutation | ΔΔG | Mutation | Kcat/Km |
| Q228A | -9.77 | N225T | -1.86 | S221R | -2.24 | V156C | 1.57 |
| T286R | -7.12 | L216R | -1.71 | T67D | -2.15 | W96N | 1.55 |
| R90D | -6.84 | Q127L | -1.53 | S169P | -2.09 | A178C | 1.54 |
| Q228G | -6.79 | Q127I | -1.44 | S188E | -2.00 | W96T | 1.54 |
| T286K | -6.55 | Q127M | -1.34 | S188F | -1.75 | W96A | 1.54 |
| S124A | -6.42 | N225S | -1.22 | S121V | -1.59 | W96S | 1.54 |
| T286H | -6.31 | R260W | -1.20 | S221M | -1.53 | M154W | 1.54 |
| T77K | -6.20 | N233R | -1.19 | A248E | -1.52 | A80C | 1.54 |
| N225A | -6.03 | A33V | -1.16 | S214M | -1.46 | F201H | 1.54 |
| T286N | -5.94 | N233A | -1.14 | S214L | -1.38 | R224F | 1.54 |

**Nucleotide sequences of the synthetic genes and expressed amino acid sequence**

**Codon-optimized nucleotide sequence of BsEstB**

ACCCATCAGATTGTGACCACGCAGTATGGCAAAGTGAAAGGCACCACCGAAAACGGCGTGCATAAATGGAAAGGCATTCCGTATGCGAAACCGCCGGTGGGTCAGTGGCGCTTTAAAGCGCCGGAACCGCCGGAAGTGTGGGAAGATGTGCTGGATGCGACCGCGTATGGCCCGATTTGCCCGCAGCCGAGCGATCTGCTGAGCCTGAGCTATACCGAACTGCCGCGTCAGAGCGAAGATTGCCTGTATGTGAACGTGTTTGCGCCGGATACCCCGAGTCAGAACCTGCCGGTGATGGTGTGGATTCATGGCGGCGCGTTTTATCTGGGCGCGGGCAGCGAACCGCTGTATGATGGCAGCAAACTGGCGGCGCAAGGCGAAGTGATTGTGGTGACCCTGAACTATCGCCTGGGCCCGTTTGGCTTTCTGCATCTGAGCAGCTTTAACGAAGCGTATAGCGATAACCTGGGCCTGCTGGATCAAGCGGCGGCGCTGAAATGGGTGCGCGAAAACATTAGCGCGTTTGGCGGCGATCCGGATAACGTGACCGTGTTTGGCGAAAGCGCGGGCGGCATGAGCATTGCGGCGCTGCTGGCGATGCCGGCGGCGAAAGGCCTGTTTCAGAAAGCGATTATGGAAAGCGGCGCGAGCCGCACCATGACCAAAGAACAAGCGGCGAGCACGAGCGCGGCGTTTCTGCAAGTGCTGGGCATTAACGAAGGTCAGCTGGATAAACTGCATACCGTGAGCGCGGAAGATAGCCTGAAGGCTGCGGATCAGCTGCGCATTGCGGAAAAAGAAAACATTTTTCAGCTGTTTTTTCAGCCGGCGCTGGATCCGAAAACCCTGCCGGAAGAACCGGAAAAAGCGATTGCGGAAGGCGCGGCGAGCGGCATTCCGCTGCTGATTGGCACCACCCGCGATGAAGGCTATCTGTTTTTTACCCCGGATAGCGATGTGCATAGCCAAGAAACCCTGGATGCGGCGCTGGAATATCTGCTGGGCAAACCGCTGGCGGAAAAGGCGGCGGATCTGTATCCGCGCAGCCTGGAAAGTCAGATTCACATGATGACCGATCTGCTGTTTTGGCGCCCGGCGGTGGCGTATGCGAGCGCGCAGAGCCATTATGCGCCGGTGTGGATGTATCGCTTTGATTGGCATCCGAAAAAACCGCCGTATAACAAAGCGTTTCATGCGCTGGAACTGCCGTTTGTGTTTGGCAACCTGGATGGCCTGGAACGCATGGCGAAAGCGGAAATTACCGATGAAGTGAAACAGCTGAGCCATACCATTCAGAGCGCGTGGATTACCTTTGCGAAAACCGGCAACCCGAGCACCGAAGCGGTGAACTGGCCGGCGTATCATGAAGAAACCCGCGAAACCCTGATTCTGGATAGCGAAATTACCATTGAAAACGATCCGGAAAGCGAAAAACGTCAGAAACTGTTTCCGAGCAAAGGCGAA

**Expressed amino acid sequence of BsEstB**

MTHQIVTTQYGKVKGTTENGVHKWKGIPYAKPPVGQWRFKAPEPPEVWEDVLDATAYGPICPQPSDLLSLSYTELPRQSEDCLYVNVFAPDTPSQNLPVMVWIHGGAFYLGAGSEPLYDGSKLAAQGEVIVVTLNYRLGPFGFLHLSSFNEAYSDNLGLLDQAAALKWVRENISAFGGDPDNVTVFGESAGGMSIAALLAMPAAKGLFQKAIMESGASRTMTKEQAASTSAAFLQVLGINEGQLDKLHTVSAEDSLKAADQLRIAEKENIFQLFFQPALDPKTLPEEPEKAIAEGAASGIPLLIGTTRDEGYLFFTPDSDVHSQETLDAALEYLLGKPLAEKAADLYPRSLESQIHMMTDLLFWRPAVAYASAQSHYAPVWMYRFDWHPKKPPYNKAFHALELPFVFGNLDGLERMAKAEITDEVKQLSHTIQSAWITFAKTGNPSTEAVNWPAYHEETRETLILDSEITIENDPESEKRQKLFPSKGELEHHHHHH

**Codon-optimized nucleotide sequence of Cut190**

GAAGTGAGCACCGCGCAAGATAACCCGTATGAACGCGGCCCGGATCCGACCGAAGATAGCATTGAAGCGATTCGCGGCCCGTTTAGCGTGGCGACCGAACGCGTGAGCAGCTTTGCGAGCGGCTTTGGCGGCGGCACCATTTATTATCCGCGCGAAACCGATGAAGGCACCTTTGGCGCGGTGGCGGTGGCGCCGGGCTTTACCGCTAGCCAAGGCAGCATGAGCTGGTATGGCGAACGCGTGGCGAGCCAAGGCTTTATTGTGTTTACCATTGATACCAACACCCGCCTGGATCAGCCGGGTCAGCGCGGCCGTCAGCTGCTGGCGGCGCTGGATTATCTGGTGGAACGCAGCGATCGCAAAGTGCGCGAACGCCTGGATCCGAACCGCCTGGCGGTGATGGGCCATAGCATGGGCGGCGGCGGCAGCCTGGAAGCGACCGTGATGCGCCCGAGCCTGAAAGCGAGCATTCCGCTGACCCCGTGGAACCTGGATAAAACCTGGGGCCAAGTGCAAGTGCCGACCTTTATTATTGGCGCGGAACTGGATACCATTGCGAGCGTGCGCACCCATGCGAAACCGTTTTATGAAAGCCTGCCGAGCAGCCTGCCGAAAGCGTATATGGAACTGGATGGCGCGACCCATTTTGCGCCGAACATTCCGAACACCACCATTGCGAAATATGTGATTAGCTGGCTGAAACGCTTTGTGGATGAAGATACCCGCTATAGTCAGTTTCTGTGCCCGAACCCGACCGATCGCGCGATTGAAGAATATCGCAGCACCTGCCCGTAT

**Expressed amino acid sequence of Cut190 (amino acid numbering starts at 39)**

M^39^EVSTAQDNPYERGPDPTEDSIEAIRGPFSVATERVSSFASGFGGGTIYYPRETDEGTFGAVAVAPGFTASQGSMSWYGERVASQGFIVFTIDTNTRLDQPGQRGRQLLAALDYLVERSDRKVRERLDPNRLAVMGHSMGGGGSLEATVMRPSLKASIPLTPWNLDKTWGQVQVPTFIIGAELDTIASVRTHAKPFYESLPSSLPKAYMELDGATHFAPNIPNTTIAKYVISWLKRFVDEDTRYSQFLCPNPTDRAIEEYRSTCPYLEHHHHHH

**Codon-optimized nucleotide sequence of *Is*PETase**

AATCCGTACGCGCGTGGTCCAAATCCAACCGCCGCGAGTCTGGAAGCGAGTGCCGGTCCATTCACCGTTCGCAGCTTTACCGTTAGCCGCCCAAGCGGTTATGGCGCCGGCACCGTGTACTACCCGACCAATGCGGGTGGCACCGTGGGTGCCATTGCGATTGTTCCGGGTTATACCGCCCGTCAGAGCAGCATCAAATGGTGGGGTCCACGTCTGGCGAGTCACGGCTTCGTGGTGATCACCATCGATACCAACAGCACGCTGGACCAGCCGAGCAGTCGCAGTAGCCAACAGATGGCCGCGCTGCGCCAAGTTGCGAGTCTGAATGGCACCAGCAGCAGCCCAATCTACGGCAAAGTGGATACCGCGCGCATGGGCGTGATGGGCTGGAGTATGGGTGGTGGCGGCAGTCTGATTAGCGCCGCCAATAACCCAAGTCTGAAAGCCGCCGCGCCACAAGCGCCGTGGGATAGTAGCACCAACTTCAGCAGTGTGACGGTTCCGACGCTGATCTTTGCGTGCGAAAACGACAGCATCGCGCCAGTGAATAGCAGCGCCCTCCCGATCTACGATAGCATGAGCCGCAATGCGAAGCAGTTTCTGGAGATCAACGGCGGTAGCCATAGCTGCGCCAACAGCGGTAACAGCAATCAAGCGCTGATCGGCAAGAAAGGCGTTGCGTGGATGAAGCGCTTCATGGACAACGATACCCGCTACAGCACGTTTGCGTGCGAGAATCCGAATAGTACGCGCGTGAGCGACTTTCGTACCGCGAATTGCAGC

**Expressed amino acid sequence of *Is*PETase (amino acid numbering starts at 27)**

M^27^GSNPYARGPNPTAASLEASAGPFTVRSFTVSRPSGYGAGTVYYPTNAGGTVGAIAIVPGYTARQSSIKWWGPRLASHGFVVITIDTNSTLDQPSSRSSQQMAALRQVASLNGTSSSPIYGKVDTARMGVMGWSMGGGGSLISAANNPSLKAAAPQAPWDSSTNFSSVTVPTLIFACENDSIAPVNSSALPIYDSMSRNAKQFLEINGGSHSCANSGNSNQALIGKKGVAWMKRFMDNDTRYSTFACENPNSTRVSDFRTANCSLEHHHHHH

**Codon-optimized nucleotide sequence of *Is*PETase-M7**

AATCCGTACGCGCGTGGTCCAAATCCAACCGCCGCGAGTCTGGAAGCGAGTGCCGGTCCATTCACCGTTCGCAGCTTTACCGTTAGCCGCCCAAGCGGTTATGGCGCCGGCACCGTGTACTACCCGACCAATGCGGGTGGCACCGTGGGTGCCATTGCGATTGTTCCGGGTTATACCGCCCGTCAGAGCAGCATCAATTGGTGGGGTCCACGTCTGGCGAGTCACGGCTTCGTGGTGATCACCATCGATACCAACAGCACGCTGGACCAGCCGAGCAGTCGCAGTAGCCAACAGATGGCCGCGCTGCGCCAAGTTGCGAGTCTGAATGGCACCAGCAGCAGCCCAATCTACGGCAAAGTGGATACCGCGCGCATGGGCGTGATGGGCTGGAGTATGGGTGGTGGCGGCAGTCTGCGTAGCGCCGCCAATAACCCAAGTCTGAAAGCCGCCGCGGTGCAAGCGCCGTGGGATAGTAGCACCAACTTCAGCAGTGTGACGGTTCCGACGCTGATCTTTGCGTGCGAAAACGACGAGATCGCGCCAGTGAATAGCGTGGCCCTCCCGATCTACGATAGCATGAGCCGCAATGCGAAGCAGTTTCTGGAGATCGGTGGCGGTAGCCATAGCTGCGCCAACAGCGGTAACAGCAATCAAGATCTGATCGGCAAGAAAGGCGTTGCGTGGATGAAGCGCTTCATGGACAACGATACCCGCTACAGCACGTTTGCGTGCGAGAATCCGAATAGTACGCGCGTGAGCGACTTTCGTACCGCGAATTGCAGC

**Expressed amino acid sequence of *Is*PETase-M7 (amino acid numbering starts at 27)**

M^27^GSNPYARGPNPTAASLEASAGPFTVRSFTVSRPSGYGAGTVYYPTNAGGTVGAIAIVPGYTARQSSINWWGPRLASHGFVVITIDTNSTLDQPSSRSSQQMAALRQVASLNGTSSSPIYGKVDTARMGVMGWSMGGGGSLRSAANNPSLKAAAVQAPWDSSTNFSSVTVPTLIFACENDEIAPVNSVALPIYDSMSRNAKQFLEIGGGSHSCANSGNSNQDLIGKKGVAWMKRFMDNDTRYSTFACENPNSTRVSDFRTANCSLEHHHHHH

**Codon-optimized nucleotide sequence of *Is*PETase-M15**

AATCCGTACGCGCGTGGTCCAAATCCAACCGCCGCGAGTCTGGAAGCGAGTGCCGGTCCATTCACCGTTCGCAGCTTTACCGTTAGCCGCCCAAGCGGTTATGGCGCCGGCACCGTGTACTACCCGACCAATGCGGGTGGCACCGTGGGTGCCATTGCGATTGTTCCGGGTTATACCGCCCGTCAGAGCAGCATCAATTGGTGGGGTCCACGTCTGGCGAGTCACGGCTTCGTGGTGATCACCATCGATACCAACAGCACGCTGGACCAGCCGAGCAGTCGCAGTAGCCAACAGATGGCCGCGCTGCGCCAAGTTGCGAGTCTGAATGGCGATAGCAGCAGCCCAATCTACGGCAAAGTGGATACCGCGCGCATGGGCGTGATGGGCTGGAGTATGGGTGGTGGCGGCAGTCTGCGTAGCGCCGCCAATAACCCAAGTCTGAAAGCCGCCGCGGTGCAAGCGCCGTGGGATAGTCGTACCGATTTCAGCAGTGTGACGGTTCCGACGCTGATCTTTGCGTGCGAAAACGACGAGATCGCGCCAGTGCAGAGCGTGGCCCTCCCGATCTACGATAGCATGAGCCGCAATGCGAAGCAGTTTCTGGAGATCGGTGGCGGTAGCCATAGCTGCGCCAACAGCGGTAACAGCAATCAAGATCTGATCGGCAAGAAAGGCGTTGCGTGGATGAAGCGCTTCATGGACAACGATACCCGCTACAGCACGTTTGCGTGCGAGAATCCGAATGATGATGAGGTGAGCGACTTTCGTACCGCGAATTGCGAG

**Expressed amino acid sequence of *Is*PETase-M15 (amino acid numbering starts at 27)**

M^27^GSNPYARGPNPTAASLEASAGPFTVRSFTVSRPSGYGAGTVYYPTNAGGTVGAIAIVPGYTARQSSINWWGPRLASHGFVVITIDTNSTLDQPSSRSSQQMAALRQVASLNGDSSSPIYGKVDTARMGVMGWSMGGGGSLRSAANNPSLKAAAVQAPWDSRTDFSSVTVPTLIFACENDEIAPVQSVALPIYDSMSRNAKQFLEIGGGSHSCANSGNSNQDLIGKKGVAWMKRFMDNDTRYSTFACENPNDDEVSDFRTANCELEHHHHHH

**Codon-optimized nucleotide sequence of *Is*PETase-DuraPETase**

AATCCGTACGCCCGTGGTCCAAATCCAACCGCGGCGAGTCTCGAAGCGAGTGCCGGCCCATTTACGGTGCGCAGCTTCACCGTGAGTCGCCCAAGCGGTTACGGTGCGGGCACCGTGTACTACCCGACCAATGCGGGTGGCACCGTTGGTGCGATCGCCATTGTGCCGGGCTACACCGCGCGCCAGAGCAGCATCAAATGGTGGGGTCCGCGTCTGGCCAGTCATGGTTTCGTGGTGATCACGATCGACACCAACAGCACGTTCGATTACCCGAGCAGTCGCAGCAGCCAACAGATGGCCGCGCTCCGTCAAGTTGCCAGTCTGAATGGCGATAGCAGCAGCCCGATTTACGGCAAGGTTGATACCGCCCGCATGGGCGTGATGGGTCACAGTATGGGTGGTGGTGCGAGTCTGCGCAGTGCGGCGAACAATCCGAGTCTGAAAGCGGCGATTCCGCAAGCCCCGTGGGATAGCCAGACGAATTTTAGCAGCGTGACGGTGCCGACCCTCATTTTCGCGTGCGAAAATGACAGCATCGCGCCGGTGAACAGTCACGCGCTGCCGATTTACGATAGCATGAGCCGCAACGCCAAGCAGTTTCTGGAAATCAACGGCGGCAGCCATAGCTGCGCGAACAGTGGTAATAGCAACCAAGCGCTGATCGGCAAGAAAGGTGTGGCGTGGATGAAGCGCTTCATGGACAACGATACCCGCTACAGCACCTTCGCGTGCGAGAACCCGAATAGTACCGCCGTGAGCGATTTTCGCACGGCGAATTGCAGC

**Expressed amino acid sequence of *Is*PETase-DuraPETase (amino acid numbering starts at 27)**

M^27^GSNPYARGPNPTAASLEASAGPFTVRSFTVSRPSGYGAGTVYYPTNAGGTVGAIAIVPGYTARQSSIKWWGPRLASHGFVVITIDTNSTLDQPSSRSSQQMAALRQVASLNGTSSSPIYGKVDTARMGVMGWSMGGGGSLISAANNPSLKAAAPQAPWDSSTNFSSVTVPTLIFACENDSIAPVNSSALPIYDSMSRNAKQFLEINGGSHSCANSGNSNQALIGKKGVAWMKRFMDNDTRYSTFACENPNSTRVSDFRTANCSLEHHHHHH

**Codon-optimized nucleotide sequence of PE-H**

AATAACCCCGCACCGACCGATCCGGGCGATAGTGGCGGCGGCTCGGCGTATCAGCGCGGCCCGGATCCGAGCGTGAGCTTTCTGGAAGCGGATCGCGGTCAGTATAGCGTGCGCAGCAGCCGCGTGAGCAGCCTGGTGAGCGGCTTTGGCGGAGGCACCATTTATTATCCGACCGGCACCACCGGCACCATGGGCGCGGTGGTGGTGATTCCGGGCTTTGTGAGCGCGGAAAGTAGCATCGATTGGTGGGGCCCGAAACTGGCGAGCTATGGCTTTGTGGTGATGACCATAGATACCAACACTGGCTTTGATCAGCCGCCGAGCCGCGCGCGTCAGATTAACAACGCGCTGGATTATCTGGTGAGTCAGAACAGCCGCAGCAGCAGCCCGGTGCGCGGCATGATAGACACCAACCGCTTGGGCGTGATTGGCTGGAGCATGGGTGGTGGAGGCACCCTTCGCGTGGCGAGCGAAGGCCGCATTAAAGCGGCGATTCCGCTGGCGCCGTGGGATACCACGAGCTATTATGCGAGCCGCAGCCAAGCGCCGACCCTGATTTTTGCGTGCGAAAGCGATGTGATTGCGCCGGTGCTGCAGCATGCGAGCCCGTTTTATAACAGCCTGCCGAGCAGCATTGATAAAGCCTTTGTGGAAATTAACGGCGGCTCTCATTATTGCGGCAATGGAGGCAGTATTTACAACGATGTGCTGAGCCGCTTTGGCGTGAGCTGGATGAAACTGCATCTGGATGAAGATAGCCGCTATAAACAGTTTCTGTGCGGCCCGAACCATACGAGCGATAGTCAGATTAGCGATTATCGCGGCAACTGCCCGTAT

**Expressed amino acid sequence of PE-H (amino acid numbering starts at 26)**

M^25^NNPAPTDPGDSGGGSAYQRGPDPSVSFLEADRGQYSVRSSRVSSLVSGFGGGTIYYPTGTTGTMGAVVVIPGFVSAESSIDWWGPKLASYGFVVMTIDTNTGFDQPPSRARQINNALDYLVSQNSRSSSPVRGMIDTNRLGVIGWSMGGGGTLRVASEGRIKAAIPLAPWDTTSYYASRSQAPTLIFACESDVIAPVLQHASPFYNSLPSSIDKAFVEINGGSHYCGNGGSIYNDVLSRFGVSWMKLHLDEDSRYKQFLCGPNHTSDSQISDYRGNCPYLEHHHHHH

**Codon-optimized nucleotide sequence of *Is*PETase-Hot**

AATCCGTACGCGCGTGGTCCAAATCCAACCGCCGCGAGTCTGGAAGCGAGTGCCGGTCCATTCACCGTTCGCAGCTTTACCGTTGCGCGCCCAGTGGGTTATGGCGCCGGCACCGTGTACTACCCGACCAATGCGGGTGGCACCGTGGGTGCCATTGCGATTGTTCCGGGTTATACCGCCACCCAGAGCAGCATCAACTGGTGGGGTCCACGTCTGGCGAGTCACGGCTTCGTGGTGATCACCATCGATACCAACAGCACGCTGGACAAACCGGAAAGTCGCAGTAGCCAACAGATGGCCGCGCTGCGCCAAGTTGCGAGTCTGAATGGCACCAGCAGCAGCCCAATCTACGGCAAAGTGGATACCGCGCGCGGTGGCGTGATGGGCTGGAGTATGGGTGGTGGCGGCAGTCTGATTAGCGCCGCCAATAACCCAAGTCTGAAAGCCGCCGCGGTGATGGCGCCGTGGCACAGTAGCACCAACTTCAGCAGTGTGACGGTTCCGACGCTGATCTTTGCGTGCGAAAACGACCGTATCGCGCCAGTGAAAGAATACGCCCTCCCGATCTACGATAGCATGAGCCTGAATGCGAAGCAGTTTCTGGAGATCTGCGGCGGTAGCCATAGCTGCGCCTGCAGCGGTAACAGCAATCAAGCGCTGATCGGCATGAAAGGCGTTGCGTGGATGAAGCGCTTCATGGACAACGATACCCGCTACAGCCAGTTTGCGTGCGAGAATCCGAATAGTACGGCGGTGTGCGACTTTCGTACCGCGAATTGCAGC

**Expressed amino acid sequence of *Is*PETase-Hot (amino acid numbering starts at 27)**

M^27^GSNPYARGPNPTAASLEASAGPFTVRSFTVARPVGYGAGTVYYPTNAGGTVGAIAIVPGYTATQSSINWWGPRLASHGFVVITIDTNSTLDKPESRSSQQMAALRQVASLNGTSSSPIYGKVDTARGGVMGWSMGGGGSLISAANNPSLKAAAVMAPWHSSTNFSSVTVPTLIFACENDRIAPVKEYALPIYDSMSLNAKQFLEICGGSHSCACSGNSNQALIGMKGVAWMKRFMDNDTRYSQFACENPNSTAVCDFRTANCSLEHHHHHH

**Codon-optimized nucleotide sequence of TurboPETase**

AGCAATCCGTATCAGCGTGGTCCGAATCCGACACGTAGCGCACTGACCACCGATGGTCCGTTTAGCGTTGCAACCTATAGCGTTAGCCGTCTGAGCGTTAGCGGTTTTGGTGGTGGTGTTATCTATTATCCGACCGGTACAACCCTGACCTTTGGTGGTATTGCAATGAGTCCGGGTTATACCGCAGATGCAAGCAGCCTGGCACTGCTGGGTCGTCGTCTGGCAAGCCATGGTTTTGTTGTTATTGTGATTAATACCAACAGCCGTCTGGATTTTCCGGATAGCCGTGCAAGCCAGCTGAGCGCAGCACTGAATTATCTGCGTACCAGCAGTCCGAGCGCAGTTCGTGCACGTCTGGATGCAAATCGTCTGGCCGTTGCAGGTCATAGCATGGGTGGTGGCGCAACCCTGCGTATTAGCGAGCAGATTCCGACACTGAAAGCCGGTGTTCCGCTGACACCGTGGCATACCGATAAAACCTTTAATACACCGGTTCCGCAGCTGATTGTTGGTGCAGAACGTGATACCGTTGCACCGGTTAGCCAGTCTGCAATTCCGATCTATCAGAATCTGCCGAGCACCACACCGAAAGTTTATGTTGAACTGAAAAATGCGACCCATACCGCACCGAATAGCCCGAATGCATGCATTAGCGTTTATACCATTAGCTGGATGAAACTGTGGGTTGATAATGATACCCGTTATCGTCAGTTTCTGTGCAATGTTAATGATCCGTGTCTGAGCGATTTTCGTAGCAATAATCGTCATTGTCAG

**Expressed amino acid sequence of TurboPETase (amino acid numbering starts at 32)**

M^32^GSSNPYQRGPNPTRSALTTDGPFSVATYSVSRLSVSGFGGGVIYYPTGTTLTFGGIAMSPGYTADASSLALLGRRLASHGFVVIVINTNSRLDFPDSRASQLSAALNYLRTSSPSAVRARLDANRLAVAGHSMGGGATLRISEQIPTLKAGVPLTPWHTDKTFNTPVPQLIVGAERDTVAPVSQSAIPIYQNLPSTTPKVYVELKNATHTAPNSPNACISVYTISWMKLWVDNDTRYRQFLCNVNDPCLSDFRSNNRHCQLEHHHHHH

**Codon-optimized nucleotide sequence of** **CaPETase^M9^**

GCCGATAACCCATACCAAAGAGGACCAGACCCAACAAATGCATCAATAGAAGCCGCAACCGGACCATTCGCAGTAGGAACACAACCAATAGTAGGAGCTTCAGGATTCGGGGGAGGACAAATATATTACCCGACCGACACCTCACAAACGTATGGCGCCGTGGTCATCGTTCCGGGCTTTATTTCCGTATGGGCACAGTTAGCGTGGTTAGGACCACGCCTGGCCAGCCAGGGTTTTGTTGTTATCGGGATTGAAACGTCTACTATTACCGATCTCCCGGATCCGCGTGGCGACCAGGCGCTGGCGGCACTGGACTGGGCGACCACACGCAGCCCCGTGAGGTCTCGCATCGATCGCACTCGCTTGGCCGCTGCCGGGTGGTCCATGGGTGGTGGCGGCCTTCGTCGTGCTGCTTGCCAACGGCCTAGTCTGAAAGCGATTGTGGGTATGGCTCCTTGGAATACCGAAAAGAACTGGAGCTGCGTCACAGTGCCGACGTTGTTCTTTGGCGGTTCAAGTGACGCAGTAGCGTCTCCGAACGATCACGCCAAACCGTTCTATAACTCGATCACCCGTGCAGAAAAAGATTACATTGAGCTGTGTAACGCCGATCATTTCTTCCCAACCTCGGCGAATACTACCATGGCGAAATACTTTATCAGCTGGCTGAAACGTTGGGTCGACAATGATACACGTTACACTCAATTCTTATGCCCTGGCCCTTCCACTGGTCTGTTTGCTCCCGTCTGTGCCTCTATGAATACCTGCCCCTTT

**Expressed amino acid sequence of CaPETase^M9^ (amino acid numbering starts at 39)**

M^39^GSADNPYQRGPDPTNASIEAATGPFAVGTQPIVGASGFGGGQIYYPTDTSQTYGAVVIVPGFISVWAQLAWLGPRLASQGFVVIGIETSTITDLPDPRGDQALAALDWATTRSPVRSRIDRTRLAAAGWSMGGGGLRRAACQRPSLKAIVGMAPWNTEKNWSCVTVPTLFFGGSSDAVASPNDHAKPFYNSITRAEKDYIELCNADHFFPTSANTTMAKYFISWLKRWVDNDTRYTQFLCPGPSTGLFAPVCASMNTCPFLEHHHHHH

**Codon-optimized nucleotide sequence of Kubu-P^M12^**

GCTGACCAAGTGGGACAAGCACCGACAGCAGCCAATATTACAGGAGATGGGTCGTTTGCGACCGCAAGCGCTCCTATTACGAATCAAACTGGTTTTGGTGGTGGTACTGTTTATTATCCTACCGCCGCGGGAACCTACCCTGTCGTAGCGGTTGTGCCGGGTTTTGTAAGCCGTTGGTCACAAATTAGTTGGCTTGGACCCCGCGTAGCCTCTTGGGGTTTTGTGGTGGTTGGTGCAGATACGAACTCTGGTTTTGATTCCCCCAGCTCTCGTGCGGATCAGCTGCTGGCCGCCCTGAATTGGGCCGTTAATTCAGCCCCCGCTGCGGTTCGTGGTAAAGTAGATGGTACCCGTCGCGGTGTAGCTGGTTGGAGCATGGGTGGTGGTGGTACACTGGAAGCATTATGCAAAGATACAACGGGTACCGTTAAAGCGGGAATCCCGCTCGCACCGTGGCACATTGGTCAAGATTTTAGTTGCGTGACGAAACCTGTATTTATTGTTGGTGCACAAAATGATACGATTGCCCCACCGGCTCAACATGCGGTTCCGTTTTATAATGCAGCAGCGGGTCCTAAATCGTACCTTGAGTTATGCGGTGCGTCTCATTTCTTTCCCACGACTGCTAATCCGACCGTTTCGCGTGCTATGGTTAGCTGGCTCAAACGTTTTGTTTCTTCAGATGATCGTTTTACACCTTTTACTTGTGGCTTTGCGGGTGCATCTGTATGCGCGTTTCGCTCGACCGCGTGT

**Expressed amino acid sequence of Kubu-P^M12^ (amino acid numbering starts at 36)**

M^36^ADQVGQAPTAANITGDGSFATASAPITNQTGFGGGTVYYPTAAGTYPVVAVVPGFVSRWSQISWLGPRVASWGFVVVGADTNSGFDSPSSRADQLLAALNWAVNSAPAAVRGKVDGTRRGVAGWSMGGGGTLEALCKDTTGTVKAGIPLAPWHIGQDFSCVTKPVFIVGAQNDTIAPPAQHAVPFYNAAAGPKSYLELCGASHFFPTTANPTVSRAMVSWLKRFVSSDDRFTPFTCGFAGASVCAFRSTACLEHHHHHH

**Codon-optimized nucleotide sequence of Mipa-P^M19^**

GCACCCCCAGCAAGTGCAACGCAGCGTGGCTGCGCACCAACGGCAGCAAATATTACGGGTGATGGTTGCTATGGTGTAGTAAGTCAGACTATTACGGGTGCGTCGGGCTTTGGCGGTGGGGTAGTTTATTATCCCAATGCTACGGAGCGTTTTGCGGTGGTTGCAATTAGCCCGGGTTATACGGAGCGTTGGAGCAGTTTTGCATGGCTGGGTCGTCGCCTCGCAAGCTGGGGCTTTGTTGTGGTGGGGATTGAAACCAATTCACTGTTTGATCAACCAAATTCGCGCGGAACTCAATTACTCCGTGCTCTCGATTGGGCGTCAACCAGCGCGCCTGCGGCAGTTCGCGATCGTGTGGATGCAACACGCCAAGGCGTAAGTGGCCATAGCATGGGTGGCGGTGGCACCCTTAGCGCGATGTGCCAACGTCCGAGCGTACGTGCTGGGGTCCCATTAGCACCTTGGCATACGACTAAATGCTGGCCCTGCGTCTGCAATCCAGTAATGATTTTGGGTGGTCAAAATGATACCATTGCCCCTGTCTCTCAGCATGCAATTCCAATGTATCAGTGCGTATGCAGTGGGGAGAAAGCCTATGTGGAACTCTGCGGTGCGGGCCATAATTTTCCGAATTCGGACAATCCCATTGTATCACGTGCTGCAGTCTCCTGGTTTAAACGCTTTCTGGATGATGATACGCGCTTTGCGCCATTTGCGTGTGATTTTGGTGGTGCGAGCATCTGCCAATTTCGTAGTACGTGCCCCGTA

**Expressed amino acid sequence of Mipa-P^M19^ (amino acid numbering starts at 37)**

M^37^APPASATQRGCAPTAANITGDGCYGVVSQTITGASGFGGGVVYYPNATERFAVVAISPGYTERWSSFAWLGRRLASWGFVVVGIETNSLFDQPNSRGTQLLRALDWASTSAPAAVRDRVDATRQGVSGHSMGGGGTLSAMCQRPSVRAGVPLAPWHTTKCWPCVCNPVMILGGQNDTIAPVSQHAIPMYQCVCSGEKAYVELCGAGHNFPNSDNPIVSRAAVSWFKRFLDDDTRFAPFACDFGGASICQFRSTCPVLEHHHHHH
